# Evaluation and Comparison of Large Language Model Responses to Frequently Asked Questions Regarding Patellofemoral Pain Syndrome: A Quality and Readability Assessment Study

**DOI:** 10.3390/healthcare14172694

**Published:** 2026-08-24

**Authors:** Oktay Polat, Berk Koncalıoğlu, Mert Gündoğdu, Emrecan Akgün

**Affiliations:** 1 Department of Orthopaedics and Traumatology, Sultanbeyli State Hospital, Istanbul 34935, Türkiye; 2 Department of Orthopaedics and Traumatology, Faculty of Medicine, Marmara University, Istanbul 34854, Türkiye

**Keywords:** patellofemoral pain syndrome, large language models, artificial intelligence, ChatGPT, Gemini, readability, DISCERN, patient education

## Abstract

**Background:** Patellofemoral pain syndrome (PFPS) is a common cause of anterior knee pain, and patients increasingly use large language models (LLMs) to obtain general medical information. However, the quality, reliability, and readability of LLM-generated responses to patient-oriented questions regarding PFPS remain uncertain. This study aimed to compare responses generated by four widely used LLMs. **Methods:** Seventeen frequently asked questions regarding PFPS were identified through Google searches and adapted into lay language. The questions were submitted to OpenAI GPT-5, Google Gemini 2.5 Pro, xAI Grok 4, and DeepSeek-V3.2-Exp using a standardized patient scenario. A total of 68 question-specific responses were independently evaluated by four orthopedic surgeons using the DISCERN instrument. Inter-rater reliability was assessed using the intraclass correlation coefficient. Readability was evaluated using the Gunning Fog Index, Coleman–Liau Index, and Flesch Reading Ease Score. Between-model comparisons were performed using the Friedman test, followed by Bonferroni-adjusted pairwise analyses. **Results:** The omnibus Friedman test showed a significant between-model difference in DISCERN scores (*p* = 0.002). In Bonferroni-adjusted pairwise comparisons, GPT-5 had lower DISCERN scores than Gemini 2.5 Pro (adjusted *p* = 0.006), Grok 4 (adjusted *p* = 0.021), and DeepSeek-V3.2-Exp (adjusted *p* = 0.036), whereas no significant differences were observed among the other three models. However, the absolute differences were small, and the between-model difference was not significant in the sensitivity analysis using the median evaluator score (*p* = 0.381). Inter-rater agreement was moderate for GPT-5 and DeepSeek-V3.2-Exp but poor for Gemini 2.5 Pro and Grok 4. Readability differed significantly among the models across all three indices. DeepSeek-V3.2-Exp generally showed more favorable numerical readability values, whereas Grok 4 tended to produce more difficult text; however, no model was consistently superior across all readability measures. The median Gunning Fog and Coleman–Liau scores for all four models exceeded the commonly recommended sixth- to eighth-grade reading level for patient education. **Conclusions:** The evaluated LLMs showed small and method-dependent differences in DISCERN-based information quality and variable differences in readability. Their responses may supplement general patient education, but the findings should not be interpreted as evidence of factual accuracy, clinical safety, or suitability for individualized decision-making. LLM-generated information should be critically reviewed and should not replace assessment by a qualified healthcare professional.

## 1. Introduction

Patellofemoral pain syndrome (PFPS) is a common cause of anterior knee pain, particularly among adolescents, young adults, and physically active individuals, and frequently prompts orthopedic consultation [1,2,3]. Its etiology is multifactorial and may involve altered lower-extremity biomechanics, muscle dysfunction, patellar maltracking, repetitive joint loading, and activity-related overuse [3,4]. Because PFPS may follow a persistent or recurrent course and its management often requires patient education, activity modification, exercise therapy, and gradual return to physical activity, patients commonly seek additional information regarding the causes, expected duration, treatment options, recurrence, and prevention of their symptoms.

Large language models (LLMs) have become increasingly accessible sources of general medical information, providing rapid and conversational responses to patient questions [5]. Models developed by different providers may help users understand medical terminology, review available management options, and obtain information presented in a more interactive format. However, the quality, reliability, and readability of LLM-generated health information may vary considerably. Responses may omit clinically relevant details, provide incomplete or unbalanced descriptions of treatment options, use language above the reading level recommended for patient education, or include unsupported statements [6,7]. These concerns are particularly relevant to PFPS because patients frequently seek guidance about exercise, physical therapy, pain relief, return to sport, recurrence, and whether surgical treatment may be necessary.

Previous studies have examined broader applications of artificial intelligence in patellofemoral disorders and the use of ChatGPT as a source of information on patellofemoral conditions [8,9]. However, direct comparisons of multiple contemporary LLMs using an identical set of PFPS-specific, patient-oriented questions remain limited. Moreover, it is unclear whether differences in DISCERN-based information quality are accompanied by corresponding differences in readability or whether orthopedic evaluators interpret the outputs of different models consistently. Therefore, this study aimed to compare the information quality and readability of responses generated by four commercially accessible LLMs—OpenAI GPT-5, Google Gemini 2.5 Pro, xAI Grok 4, and DeepSeek-V3.2-Exp—to 17 frequently asked questions regarding PFPS. Each question-specific response was assessed using the DISCERN instrument and three established readability indices, and inter-rater agreement among the orthopedic surgeon evaluators was also examined.

## 2. Materials and Methods

### 2.1. Study Design and Ethical Considerations

This cross-sectional observational study was conducted between September and November 2025. The study was based exclusively on textual responses generated by LLMs for a generic patient scenario. No patients, patient records, identifiable personal health information, medical images, biological materials, or clinical interventions were involved. No patient-specific medical history or confidential information was entered into any of the evaluated models.

The responses were assessed by four orthopedic surgeons who were members of the research team and authors of the manuscript. Their professional characteristics were reported descriptively to provide context for the evaluation and were not analyzed as study outcomes. Because the study did not involve patients, patient data, biological materials, or clinical interventions, ethics committee approval and patient informed consent were considered not applicable.

### 2.2. Preparation of Questions and LLM-Generated Responses

Frequently asked questions concerning PFPS were identified through structured Google searches conducted in English during the study period. The search terms, patient-oriented topics identified, and their mapping to the final question set are presented in Supplementary Table S1. Questions were eligible when they addressed information commonly sought by patients regarding the definition, causes, symptoms, treatment, prognosis, physical activity, return to sport, recurrence, or prevention of PFPS. Questions requiring interpretation of patient-specific examination findings, medical records, or imaging studies were excluded.

The identified questions were independently reviewed by four orthopedic surgeons and revised into lay language without changing their intended clinical meaning. Duplicate questions and semantically overlapping formulations were merged by consensus. This process resulted in 17 final questions, each of which was assigned an identification code from Q1 to Q17 to permit direct mapping between the questions and the corresponding model responses.

Four commercially accessible LLMs developed by different providers were evaluated: OpenAI GPT-5, Google Gemini 2.5 Pro, xAI Grok 4, and DeepSeek-V3.2-Exp. These models were selected because they represented contemporary and widely accessible LLM systems developed by four different providers at the time of data collection, thereby allowing a cross-provider comparison. GPT-5 was accessed through the paid ChatGPT web interface, Gemini 2.5 Pro through a paid Gemini account, Grok 4 through a paid SuperGrok subscription, and DeepSeek-V3.2-Exp through a paid DeepSeek account. The developer, model version, access platform, subscription status, exact access date, and query settings for each model are presented in Supplementary Table S2.

A separate chat session was opened for each model. The same standardized patient scenario and the complete set of 17 questions were submitted together to each model in a single prompt using identical wording and question order. Default user-facing settings were used. No optional thinking, extended-reasoning, or enhanced-reasoning mode was manually activated. GPT-5, Gemini 2.5 Pro, and Grok 4 were queried using their standard default interfaces, whereas DeepSeek-V3.2-Exp was queried using its Fast mode rather than its reasoning mode. Only the initial combined response generated by each model was retained. No follow-up prompts, regeneration requests, clarification prompts, or manual modifications were applied.

The standardized prompt began with the following patient scenario:

“I went to the orthopedic outpatient clinic because of knee pain. I was told that I have patellofemoral pain syndrome, and I have a few questions about this condition.”

The scenario was immediately followed by the same 17 patient-oriented questions listed in Supplementary Table S1. The models were not provided with patient-specific information and were not asked to establish an individualized diagnosis, select a patient-specific treatment, or determine whether a particular patient required surgery. The complete initial responses generated by the four models are provided in Part S1 of the Supplementary File.

**Figure 1:**
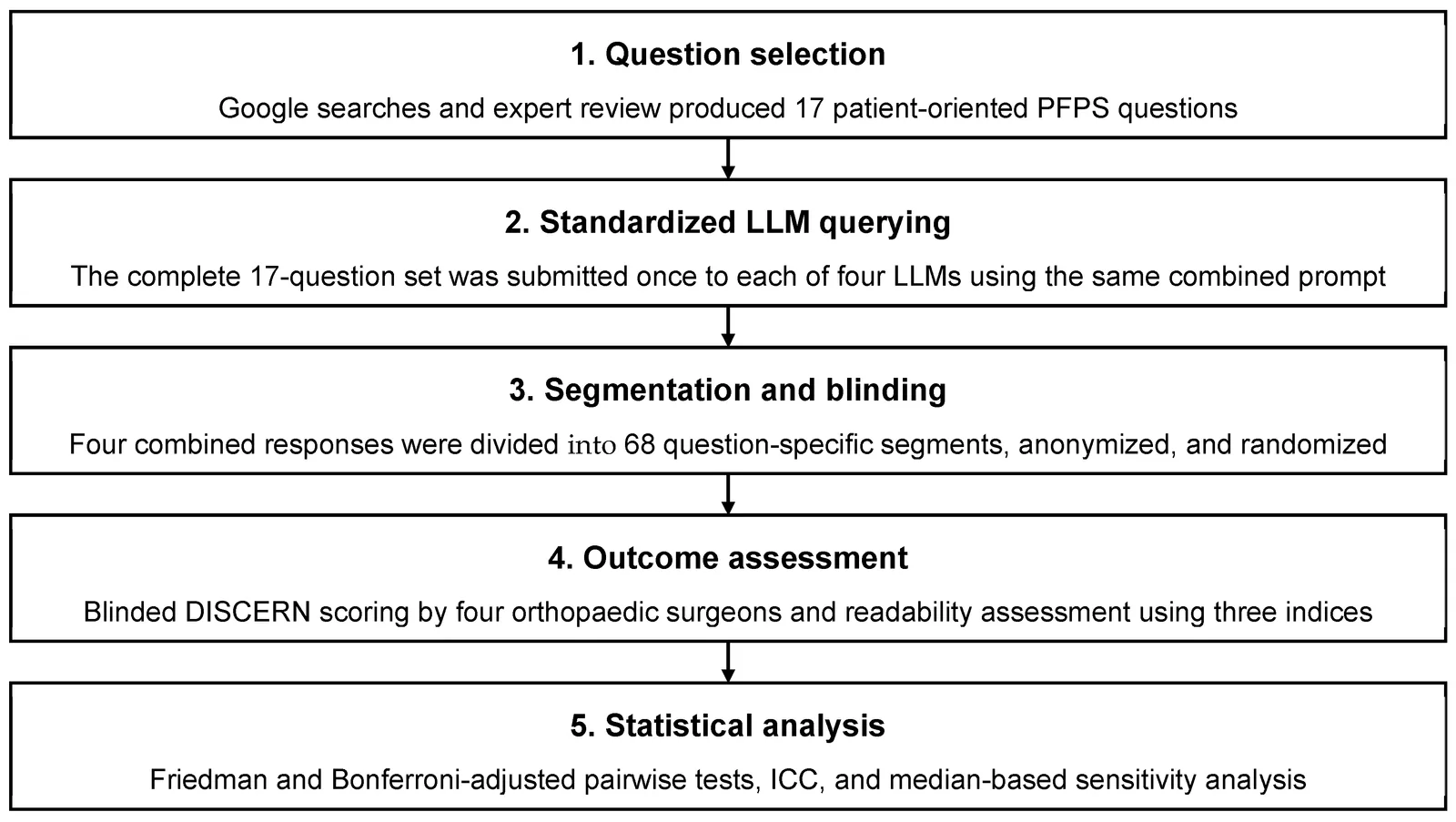
Study workflow.

The overall study process, including question identification, model querying, anonymization, randomized evaluation, readability assessment, and statistical analysis, is summarized in Figure 1.

Patient-oriented questions regarding patellofemoral pain syndrome were identified through Google searches, reviewed, and consolidated into a final set of 17 questions. The complete question set was submitted to each of four large language models in a single standardized prompt. The four combined responses were divided into 68 question-specific answer segments, which were anonymized and randomized before blinded, individual DISCERN scoring by four orthopedic surgeons. Readability was assessed using the Gunning Fog Index, Coleman–Liau Index, and Flesch Reading Ease Score, followed by statistical comparison across the models.

### 2.3. Evaluation of the LLM-Generated Responses

Each combined model response was divided into answer segments corresponding to the predefined questions using the Q1–Q17 identification system. The answer segments were exported and assigned anonymized identification codes. Model names and any other information that could reveal the generating model were removed. The anonymized question-specific answer segments were then presented to the evaluators in a randomized order.

The answer segments were independently evaluated by four orthopedic surgeons who were blinded to the identity of the generating model and to the scores assigned by the other evaluators. No formal calibration exercise, consensus scoring session, or discussion of individual ratings was conducted before or during the formal evaluation. All four evaluators were members of the research team and authors of the manuscript. Their clinical experience, experience in managing PFPS, and previous use of LLMs are summarized descriptively in Supplementary Table S3.

Each answer segment corresponding to each of the 17 questions was evaluated separately. The unit of analysis was therefore the individual question-specific answer segment rather than the complete combined response generated by a model. A total of 68 question-specific answer segments were evaluated, comprising 17 answer segments from each of the four models.

### 2.4. DISCERN Assessment

The quality and reliability of the responses were assessed using the 16-item DISCERN instrument [10], a structured tool used to evaluate the quality of patient-oriented health information concerning treatment choices. No DISCERN items or scoring categories were modified; however, applying the instrument to individual question-specific chatbot responses represents an adaptation of its conventional use.

DISCERN includes 16 items, each scored on a 5-point scale, and produces a total score ranging from 16 to 80. Higher scores indicate better quality, reliability, balance, and usefulness of the presented information. Each evaluator assigned a separate total DISCERN score to every question-specific response.

DISCERN does not independently establish the factual or clinical accuracy of individual statements. The study therefore evaluated DISCERN-based information quality and reliability rather than factual accuracy. No separate item-by-item assessment of factual errors, clinically important omissions, unsupported statements, or potentially harmful recommendations was performed.

The anonymized total DISCERN score dataset for the four evaluators is provided in Part S2 of the Supplementary File.

### 2.5. Readability Assessment

Readability was assessed separately for each question-specific response using the Gunning Fog Index, Coleman–Liau Index, and Flesch Reading Ease Score. The Gunning Fog Index estimates the number of years of formal education required to understand a text, with higher values indicating greater reading difficulty [11]. The Coleman–Liau Index estimates the corresponding United States grade level based on the number of characters, words, and sentences in the text [12]. The Flesch Reading Ease Score evaluates readability according to sentence length and word complexity, with higher values indicating easier-to-read content [13].

Patient education materials are generally recommended to be written at approximately a sixth- to eighth-grade reading level to facilitate comprehension among a broad audience [14,15].

Readability was calculated separately for each of the 17 question-specific responses generated by each model. Consequently, 17 paired observations were available for each model and each readability index. This approach ensured that the DISCERN and readability analyses used the same unit of analysis. Responses generated by each model were not combined into a single continuous text.

### 2.6. Statistical Analysis

Statistical analyses were performed using IBM SPSS Statistics, version 31.0.0.0 (IBM Corp., Armonk, NY, USA). The distribution of numerical variables was evaluated using the Shapiro–Wilk test and visual inspection of the distributions. Because several variables did not satisfy the assumption of normality and the observations were paired by question, DISCERN and readability results were summarized as medians with interquartile ranges and analyzed using nonparametric methods.

For the primary DISCERN analysis, the mean score assigned by the four evaluators was calculated for each question-specific response. This yielded 17 paired DISCERN observations for each model.

Differences among the four models were evaluated using the Friedman test. When the omnibus Friedman test was statistically significant, post hoc pairwise comparisons were conducted using the Wilcoxon signed-rank test. Bonferroni-adjusted *p* values were reported to account for the six possible pairwise comparisons. The Friedman chi-square statistic, degrees of freedom, *p* value, and Kendall’s W effect size were reported.

Inter-rater reliability among the four evaluators was assessed separately for each model using a two-way random-effects model with a consistency definition and the average-measures intraclass correlation coefficient (ICC). The average-measures ICC was selected because the primary DISCERN analysis was based on the mean score assigned by the four evaluators.

ICC values were interpreted as poor when below 0.50, moderate between 0.50 and 0.75, good between 0.75 and 0.90, and excellent when above 0.90 [16]. Negative ICC values were retained and interpreted as indicating that disagreement among the evaluators exceeded the variability among the assessed responses.

A sensitivity analysis was conducted using the median rather than the mean of the four evaluator scores for each question-specific response. This analysis examined whether the between-model comparison was sensitive to differences in the way individual evaluators applied the DISCERN criteria.

No a priori sample-size calculation was performed because the study included the complete set of 17 predefined frequently asked questions. The exploratory design and limited number of question-specific observations were considered when interpreting nonsignificant findings. All statistical tests were two-sided, and *p* < 0.05 was considered statistically significant.

## 3. Results

For each question-specific response, the mean DISCERN score assigned by the four evaluators was calculated and used in the primary comparison. The median DISCERN scores were 53.50 (50.00–55.25) for GPT-5, 55.25 (51.50–57.25) for Gemini 2.5 Pro, 55.00 (54.25–56.75) for Grok 4, and 54.75 (51.00–57.50) for DeepSeek-V3.2-Exp. The Friedman test showed a statistically significant difference among the four models [χ^2^(3) = 14.360, *p* = 0.002, Kendall’s W = 0.282] (Table 1).

A total of 68 question-specific responses, comprising 17 responses from each of the four models, were included in the DISCERN and readability analyses. Inter-rater reliability differed across the models. Moderate agreement was observed for OpenAI GPT-5 (ICC = 0.733, 95% CI: 0.442–0.892) and DeepSeek-V3.2-Exp (ICC = 0.666, 95% CI: 0.301–0.865), whereas agreement was poor for Google Gemini 2.5 Pro (ICC = 0.420, 95% CI: −0.214–0.766) and xAI Grok 4 (ICC = −0.304, 95% CI: −1.730–0.474) (Table 2). The negative ICC for Grok 4 indicated that disagreement among the evaluators exceeded the variability among the evaluated responses.

Post hoc comparisons with Bonferroni adjustment showed that GPT-5 had lower DISCERN scores than Gemini 2.5 Pro (adjusted *p* = 0.006), Grok 4 (adjusted *p* = 0.021), and DeepSeek-V3.2-Exp (adjusted *p* = 0.036). No significant differences were observed between Gemini 2.5 Pro and Grok 4 (adjusted *p* = 1.000), Gemini 2.5 Pro and DeepSeek-V3.2-Exp (adjusted *p* = 1.000), or Grok 4 and DeepSeek-V3.2-Exp (adjusted *p* = 0.526). Although the comparisons involving GPT-5 were statistically significant, the absolute differences in median DISCERN scores were small.

In the sensitivity analysis, the median rather than the mean of the four evaluator scores was calculated for each question-specific response. Under this approach, the difference among the four models was no longer statistically significant [χ^2^(3) = 3.072, *p* = 0.381, Kendall’s W = 0.060]. This finding indicated that the primary between-model comparison was sensitive to the method used to summarize the evaluator scores.

**Figure 2:**
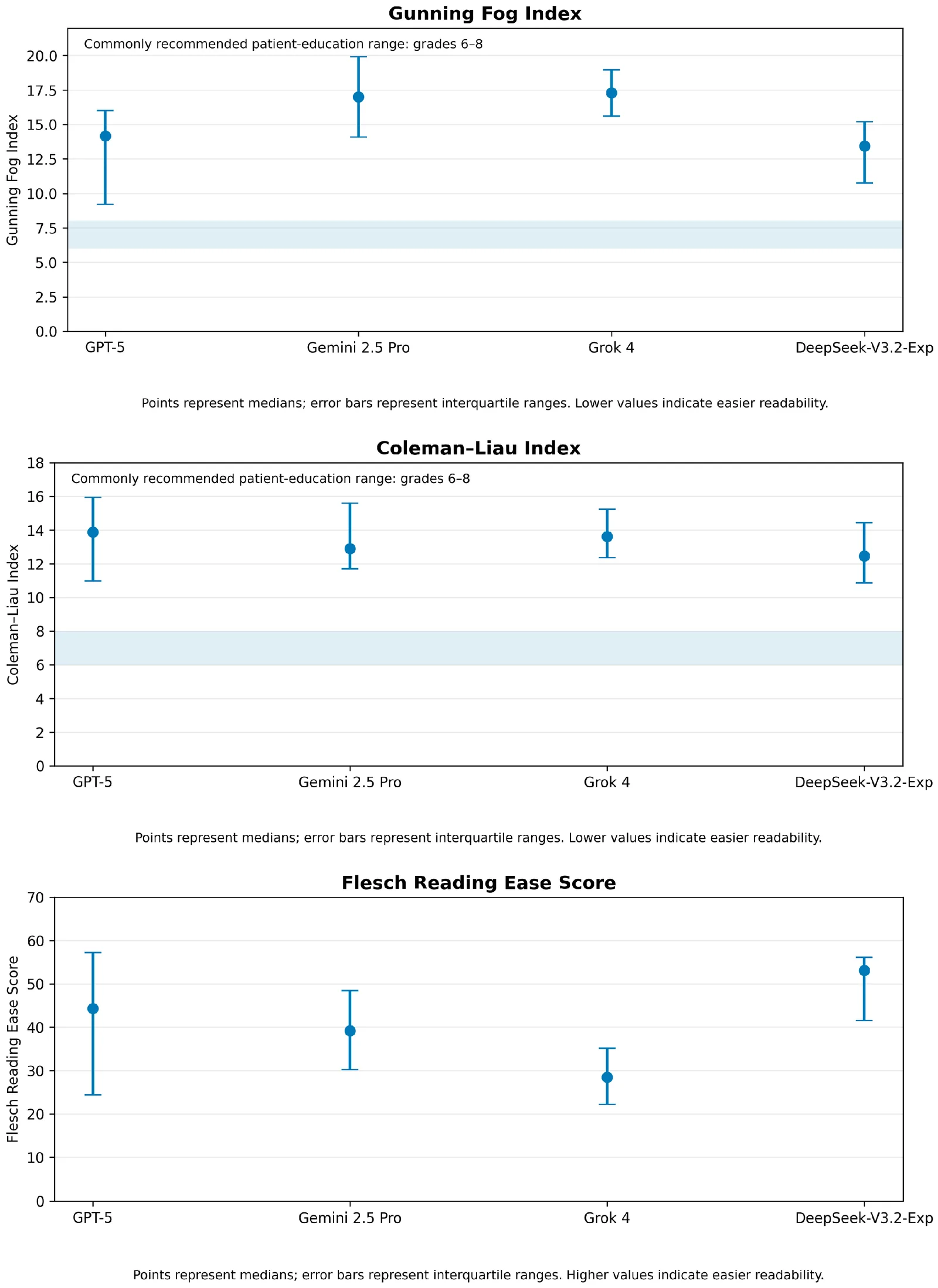
**Comparison of readability measures across the evaluated large language models.** Points represent median values, and error bars represent interquartile ranges based on 17 question-specific responses per model. Lower Gunning Fog Index and Coleman–Liau Index values indicate easier readability, whereas higher Flesch Reading Ease Scores indicate easier readability. The shaded areas in the Gunning Fog and Coleman–Liau panels indicate the commonly recommended sixth- to eighth-grade reading level for patient education materials.

Readability was assessed separately for each of the 17 question-specific responses generated by each model. Significant differences were observed for all three readability indices (Table 3), and the median and interquartile ranges of the readability measures across the four models are illustrated in Figure 2.

For the Gunning Fog Index, the median scores were 14.17 (9.22–16.00) for GPT-5, 17.00 (14.10–19.90) for Gemini 2.5 Pro, 17.29 (15.60–18.96) for Grok 4, and 13.43 (10.76–15.20) for DeepSeek-V3.2-Exp [χ^2^(3) = 20.929, *p* < 0.001, Kendall’s W = 0.410]. GPT-5 had lower scores than Gemini 2.5 Pro (adjusted *p* = 0.014) and Grok 4 (adjusted *p* = 0.012), whereas DeepSeek-V3.2-Exp had lower scores than Gemini 2.5 Pro (adjusted *p* = 0.014) and Grok 4 (adjusted *p* = 0.004). No significant difference was observed between GPT-5 and DeepSeek-V3.2-Exp. The complete post hoc comparisons are presented in Table 4.

For the Coleman–Liau Index, the median scores were 13.88 (10.98–15.93) for GPT-5, 12.90 (11.70–15.60) for Gemini 2.5 Pro, 13.62 (12.36–15.23) for Grok 4, and 12.45 (10.86–14.43) for DeepSeek-V3.2-Exp [χ^2^(3) = 8.082, *p* = 0.044, Kendall’s W = 0.158]. After Bonferroni adjustment, only the difference between Grok 4 and DeepSeek-V3.2-Exp remained statistically significant (adjusted *p* = 0.008).

For the Flesch Reading Ease Score, the median values were 44.32 (24.44–57.18) for GPT-5, 39.21 (30.22–48.40) for Gemini 2.5 Pro, 28.41 (22.12–35.10) for Grok 4, and 53.08 (41.51–56.14) for DeepSeek-V3.2-Exp [χ^2^(3) = 12.459, *p* = 0.006, Kendall’s W = 0.244]. Grok 4 had lower scores than Gemini 2.5 Pro (adjusted *p* = 0.039) and DeepSeek-V3.2-Exp (adjusted *p* = 0.007). The difference between GPT-5 and Grok 4 did not remain statistically significant after adjustment (adjusted *p* = 0.052).

DeepSeek-V3.2-Exp generally showed numerically more favorable readability values, whereas Grok 4 tended to produce more difficult text. However, no model was consistently superior across all three readability measures, and the direction and statistical significance of the pairwise differences varied according to the formula used. Median Gunning Fog and Coleman–Liau scores for all four models remained above the commonly recommended sixth- to eighth-grade reading level for patient education materials.

## 4. Discussion

The present study compared the DISCERN-based information quality and readability of responses generated by four LLMs to 17 patient-oriented questions regarding PFPS. The principal findings were that OpenAI GPT-5 received statistically lower DISCERN scores than Google Gemini 2.5 Pro, xAI Grok 4, and DeepSeek-V3.2-Exp, although the absolute differences among the models were small. Readability also varied among the models, but the direction and magnitude of the pairwise differences depended on the readability formula applied. Inter-rater agreement was moderate for GPT-5 and DeepSeek-V3.2-Exp but poor for Gemini 2.5 Pro and Grok 4, indicating that the DISCERN comparisons should be interpreted cautiously. Moreover, the primary between-model difference was not reproduced when the median rather than the mean of the four evaluator scores was used.

The overall difference in DISCERN scores was statistically significant, and Kendall’s W indicated a small-to-moderate between-model effect. GPT-5 received lower scores than each of the other three models, whereas Gemini 2.5 Pro, Grok 4, and DeepSeek-V3.2-Exp did not differ significantly from one another. However, the difference between the highest and lowest median DISCERN scores was less than two points on a scale ranging from 16 to 80. Furthermore, no established threshold defines a clinically or practically meaningful difference in DISCERN scores for LLM-generated information. Therefore, the statistically significant primary result should not be interpreted as evidence that patients would necessarily receive meaningfully different information from the four models.

The sensitivity analysis further reduced confidence in a definitive ranking of the models. When the median score of the four evaluators was used for each question-specific response, the overall difference was no longer statistically significant [χ^2^(3) = 3.072, *p* = 0.381, Kendall’s W = 0.060]. This suggests that the primary comparison was influenced, at least partly, by differences in how individual evaluators applied the DISCERN criteria. The observed ordering of the models should therefore be considered exploratory rather than conclusive.

Patel et al. [17] also reported model-dependent variation in DISCERN and readability outcomes when evaluating chatbot-generated information on bladder cancer. However, the relative performance of the models in their study differed from that observed in the present analysis. Comparisons across studies should be made cautiously because LLM performance may be influenced by the clinical topic, complexity and wording of the questions, prompt structure, model version, access mode, and date of evaluation. Findings obtained in an oncological information setting may therefore not be directly transferable to patient questions concerning a musculoskeletal condition.

Comparative studies in other clinical fields have similarly demonstrated heterogeneous performance among LLMs and have identified clinically relevant errors or omissions, supporting the need for professional review [18]. Twomey-Kozak et al. [8] systematically reviewed the diagnostic and predictive performance of artificial intelligence in patellofemoral osteoarthritis, trochlear dysplasia, and patellofemoral tracking abnormalities. Their review focused mainly on imaging-based diagnostic and predictive applications rather than on the quality and readability of patient-oriented LLM responses. Frodl et al. [9] evaluated ChatGPT as a source of information on patellofemoral conditions and reported that orthopedic specialists rated responses to more complex questions less favorably than non-experts. This finding suggests that evaluations of LLM-generated information may be influenced by the clinical background and expectations of the evaluator, in addition to the characteristics of the response itself.

Inter-rater agreement was not consistent across the evaluated models. Agreement was moderate for GPT-5 and DeepSeek-V3.2-Exp but poor for Gemini 2.5 Pro and Grok 4. These findings do not demonstrate that any individual model generated inaccurate or conceptually ambiguous responses. Rather, they indicate that the evaluators applied the DISCERN criteria less consistently to the responses generated by some models. Differences in response length, organization, level of detail, discussion of uncertainty, or presentation of treatment alternatives may have contributed to this variation. However, these possibilities were not examined directly, and no causal explanation for the lower ICC values can be established. Comparisons involving Gemini 2.5 Pro and Grok 4 should therefore be interpreted with particular caution.

DISCERN is a structured instrument used to evaluate the quality of written consumer health information concerning treatment choices [10]. It assesses features such as transparency, balance, discussion of treatment alternatives, risks, benefits, and support for shared decision-making, but it does not independently determine whether each medical statement is factually correct. Accordingly, the present findings describe differences in information quality and presentation as measured by DISCERN rather than differences in clinical accuracy. Claims that the evaluated models produced accurate, clinically acceptable, or safe information would exceed what can be supported by the present methodology. A separate expert assessment of factual errors, clinically important omissions, unsupported statements, and potentially harmful recommendations would be required to evaluate clinical accuracy and safety.

Readability is an additional concern when model-generated responses are intended for patient education. Previous studies have reported that LLM-generated patient information frequently exceeds the reading level recommended for the general population [7,19,20]. Carlson et al. [21] similarly observed differences in readability among chatbot responses even when their medical content was otherwise comparable. In the present study, DeepSeek-V3.2-Exp generally showed the most favorable numerical readability values, whereas Grok 4 tended to generate more difficult text. However, DeepSeek-V3.2-Exp was not significantly easier to read than every other model across all three indices. For example, GPT-5 and DeepSeek-V3.2-Exp showed similar Gunning Fog values, and several pairwise comparisons did not remain significant after adjustment for multiple testing. The findings therefore do not demonstrate the consistent readability superiority of a single model.

Despite the observed differences, the median Gunning Fog and Coleman–Liau values of all four models exceeded the commonly recommended sixth- to eighth-grade reading level for patient education materials [14,15]. Thus, even responses with comparatively favorable readability scores may remain difficult for some patients to understand. Readability formulas estimate textual difficulty using characteristics such as sentence length, word length, syllable count, and character count. They do not assess conceptual understanding, numeracy, cultural appropriateness, prior medical knowledge, or a patient’s ability to apply the information to a personal health decision. Because patients and non-medical readers were not included as evaluators, the actual comprehensibility and usefulness of the responses in routine practice remain uncertain.

The findings should also be interpreted within the limited scope of the standardized scenario used in this study. The models responded to generic questions and were not provided with patient records, medical histories, physical examination findings, imaging results, or identifiable personal information. The study therefore did not evaluate the use of LLMs for individualized diagnosis, treatment selection, or determination of surgical indications. Given continuing concerns regarding privacy and reliability in complex health-related tasks, users should avoid entering confidential health information into commercially available LLM interfaces, and model-generated responses should not replace clinical assessment or professional medical advice [22].

Several limitations should be considered. First, this study represents a cross-sectional evaluation of specific model versions accessed October 2025. Because LLMs are updated frequently, the findings may not apply to earlier or later versions of the same models. Second, each standardized question set was submitted only once to each model. The analysis therefore did not capture within-model variability across repeated generations, and different responses might have been produced if the same questions had been submitted again.

Third, the analysis was limited to English-language responses and a single musculoskeletal condition. The findings may not be generalizable to other languages, clinical specialties, patient populations, or types of medical questions.

Fourth, the same research team participated in identifying and refining the questions and subsequently evaluated the model-generated responses. This closed-loop design may have introduced question-selection bias, observer-expectancy bias, confirmation bias, and shared interpretative bias arising from the evaluators’ similar professional backgrounds. Although model identifiers were removed, response order was randomized, and scoring was performed independently, these precautions cannot completely eliminate such biases. External clinicians who were not involved in question development, patients, and non-medical readers were not included; therefore, independent external validation was lacking. Future studies should incorporate independently developed or externally validated question sets and include these evaluator groups.

Fifth, inter-rater agreement was poor for some models, reducing confidence in the stability of the corresponding DISCERN comparisons. The nonsignificant sensitivity analysis also demonstrated that the primary result depended partly on whether evaluator scores were summarized using the mean or median. Sixth, DISCERN was applied to individual conversational responses, although the instrument is intended for written consumer health materials. Although none of its items were modified, its application to question-specific chatbot responses represents an adaptation of its conventional use.

Seventh, factual accuracy, clinically important omissions, unsupported statements, and potentially harmful recommendations were not evaluated using a separate validated framework. Eighth, no a priori sample-size calculation was performed, and the analysis was based on the complete set of 17 predefined questions. The limited number of paired observations may have reduced the ability to detect small differences and increased the influence of individual questions on the overall findings. Finally, readability formulas provide indirect estimates of textual complexity and cannot determine whether patients actually understood, trusted, or acted appropriately on the information provided.

## 5. Conclusions

The four evaluated models showed small and method-dependent differences in DISCERN-based information quality and more variable differences in readability. GPT-5 received statistically lower DISCERN scores than Gemini 2.5 Pro, Grok 4, and DeepSeek-V3.2-Exp in the primary analysis. However, the absolute differences were small, their practical significance remains uncertain, and the between-model difference was not significant in the median-based sensitivity analysis.

DeepSeek-V3.2-Exp generally produced more favorable numerical readability values, whereas Grok 4 tended to generate more difficult text. Nevertheless, no model was consistently superior across all three readability measures, and the median Gunning Fog and Coleman–Liau scores for all four models remained above the reading level commonly recommended for patient education.

These findings represent a time-specific evaluation of English-language responses to PFPS-related questions and should not be interpreted as evidence of factual accuracy, clinical safety, or suitability for individualized decision-making. LLM-generated information may supplement general patient education, but it requires critical review and should not replace assessment by a qualified healthcare professional. Future studies should include repeated model sampling, independent evaluation of factual accuracy and potentially harmful content, patient and non-medical evaluator feedback, and comparisons across languages, clinical conditions, and updated model versions.

## Figures and Tables

**Table 1:** Comparison of DISCERN scores among the large language models.

**Panel A. Overall DISCERN Scores**			
**Model**		**DISCERN Score, Median (IQR)**	
**OpenAI GPT-5**		53.50 (50.00–55.25)	
**Google Gemini 2.5 Pro**		55.25 (51.50–57.25)	
**xAI Grok 4**		55.00 (54.25–56.75)	
**DeepSeek-V3.2-Exp**		54.75 (51.00–57.50)	
**Panel B. Post hoc pairwise comparisons**			
**Pairwise comparison**		**Bonferroni-adjusted *p* value**	
GPT-5 vs. Gemini 2.5 Pro		0.006	
GPT-5 vs. Grok 4		0.021	
GPT-5 vs. DeepSeek-V3.2-Exp		0.036	
Gemini 2.5 Pro vs. Grok 4		1.000	
Gemini 2.5 Pro vs. DeepSeek-V3.2-Exp		1.000	
Grok 4 vs. DeepSeek-V3.2-Exp		0.526	
**Panel C. Sensitivity analysis**			
Analysis	Friedman χ^2^	*p* value	Kendall’s W
Median of the four evaluator scores	3.072	0.381	0.060

Friedman χ^2^(3) = 14.360; *p* = 0.002; Kendall’s W = 0.282. Values in Panel A are presented as median (interquartile range) based on 17 question-specific observations per model. For each question-specific response, the mean DISCERN score assigned by the four evaluators was used in the primary analysis. Pairwise comparisons were performed using the Wilcoxon signed-rank test, with Bonferroni adjustment for six comparisons. The sensitivity analysis was based on the median rather than the mean of the four evaluator scores. DISCERN, quality assessment instrument for written consumer health information; IQR, interquartile range.

**Table 2:** Inter-rater reliability of DISCERN evaluations for each large language model.

Model	ICC	95% CI	*p* Value	Interpretation
OpenAI GPT-5	0.733	0.442 to 0.892	<0.001	Moderate
Google Gemini 2.5 Pro	0.420	−0.214 to 0.766	0.074	Poor
xAI Grok 4	−0.304	−1.730 to 0.474	0.713	Poor
DeepSeek-V3.2-Exp	0.666	0.301 to 0.865	0.002	Moderate

ICC, intraclass correlation coefficient; CI, confidence interval. Inter-rater reliability was calculated separately for each model across the 17 question-specific responses using a two-way random-effects model, a consistency definition, and the average-measures ICC. ICC values were interpreted as poor (<0.50), moderate (0.50–0.75), good (0.75–0.90), or excellent (>0.90).

**Table 3:** Comparison of readability scores among the large language models.

Readability Measure	OpenAI GPT-5	Google Gemini 2.5 Pro	xAI Grok 4	DeepSeek-V3.2-Exp	Friedman χ^2^	*p* Value	Kendall’s W
Gunning Fog Index	14.17 (9.22–16.00)	17.00 (14.10–19.90)	17.29 (15.60–18.96)	13.43 (10.76–15.20)	20.929	<0.001	0.410
Coleman–Liau Index	13.88 (10.98–15.93)	12.90 (11.70–15.60)	13.62 (12.36–15.23)	12.45 (10.86–14.43)	8.082	0.044	0.158
Flesch Reading Ease Score	44.32 (24.44–57.18)	39.21 (30.22–48.40)	28.41 (22.12–35.10)	53.08 (41.51–56.14)	12.459	0.006	0.244

Values are presented as median (interquartile range) based on 17 question-specific responses per model. Higher Gunning Fog and Coleman–Liau scores indicate greater reading difficulty, whereas higher Flesch Reading Ease scores indicate easier readability. Comparisons among the four models were performed using the Friedman test. Kendall’s W represents the effect size.

**Table 4:** Post hoc pairwise comparisons of readability scores.

Readability Measure	Pairwise Comparison	Bonferroni-Adjusted *p* Value
Gunning Fog Index	GPT-5 vs. Gemini 2.5 Pro	0.014
	GPT-5 vs. Grok 4	0.012
	GPT-5 vs. DeepSeek-V3.2-Exp	1.000
	Gemini 2.5 Pro vs. Grok 4	1.000
	Gemini 2.5 Pro vs. DeepSeek-V3.2-Exp	0.014
	Grok 4 vs. DeepSeek-V3.2-Exp	0.004
Coleman–Liau Index	GPT-5 vs. Gemini 2.5 Pro	1.000
	GPT-5 vs. Grok 4	1.000
	GPT-5 vs. DeepSeek-V3.2-Exp	1.000
	Gemini 2.5 Pro vs. Grok 4	1.000
	Gemini 2.5 Pro vs. DeepSeek-V3.2-Exp	1.000
	Grok 4 vs. **DeepSeek-V3.2-Exp**	0.008
Flesch Reading Ease Score	GPT-5 vs. Gemini 2.5 Pro	1.000
	GPT-5 vs. Grok 4	0.052
	GPT-5 vs. **DeepSeek-V3.2-Exp**	1.000
	Gemini 2.5 Pro vs. Grok 4	0.039
	Gemini 2.5 Pro vs. DeepSeek-V3.2-Exp	1.000
	Grok 4 vs. DeepSeek-V3.2-Exp	0.007

Pairwise comparisons were performed using the Wilcoxon signed-rank test. Reported *p* values were adjusted for six comparisons using the Bonferroni method.

## Data Availability

The complete LLM-generated responses and the anonymized total DISCERN score dataset are provided in Parts S1 and S2, respectively, of a single combined Supplementary Materials file. Additional data are available from the corresponding author upon reasonable request.

## Supplementary Materials

The following supporting information can be downloaded at: https://www.mdpi.com/article/10.3390/healthcare14172694/s1, Table S1: Google search strategy and mapping of patient-oriented topics to the final question set; Table S2: Access details and query settings of the evaluated large language models; Table S3: Characteristics of the evaluators; Part S1: Complete initial responses generated by the four large language models; Part S2: Anonymized total DISCERN score dataset for the four evaluators.

## Abbreviations

AAOS: American Academy of Orthopaedic Surgeons, AI: Artificial intelligence, CI: Confidence interval, DISCERN: Quality assessment instrument for consumer health information, ICC: Intraclass correlation coefficient, LLM: Large language model, IQR: Interquartile range, PFPS: Patellofemoral pain syndrome.
