# Supplementary material for: Evaluation and Comparison of Large Language Model Responses to Frequently Asked Questions Regarding Patellofemoral Pain Syndrome: A Quality and Readability Assessment Study
Source: Healthcare (Basel). 2026 Aug 24;14(17):2694. doi: 10.3390/healthcare14172694 (PMC13565727; doi:10.3390/healthcare14172694)
Supplement: Supplementary file 1 [file healthcare-14-02694-s001.zip › healthcare-4434527-supplementary.pdf]

COMBINED SUPPLEMENTARY MATERIAL

for the manuscript

Evaluation and comparison of large language model responses to frequently asked questions regarding patellofemoral pain syndrome: a quality and readability assessment study

Oktay Polat, Berk Koncalıoğlu, Mert Gündoğdu, and Emrecan Akgün

Contents

1. Table S1: Google search strategy and mapping of patient-oriented topics to the final question set

2. Table S2: Access details and query settings of the evaluated large language models

3. Table S3: Characteristics of the evaluators

4. Part S1: Complete initial responses generated by the four large language models

5. Part S2: Anonymized total DISCERN score dataset for the four evaluators

Supplementary Table S1. Google search strategy and mapping of patient-oriented topics to the final question set.

Search Period	Search Term Used	Patient-Oriented Topic Identified	Final Question Code	Final Question
October 2025	“patellofemoral pain syndrome frequently asked questions”	General definition and commonly asked questions	Q1	What exactly is patellofemoral pain syndrome?
October 2025	“what causes patellofemoral pain syndrome knee pain”	Etiology and contributing factors	Q2	What causes my pain?
October 2025	“runner’s knee and patellofemoral pain syndrome same condition”	Alternative terminology	Q3	Is this the same as “runner’s knee” or patellofemoral syndrome?
October 2025	“activities that worsen patellofemoral pain syndrome”	Aggravating activities	Q4	What activities make the pain worse?
October 2025	“patellofemoral pain syndrome arthritis cartilage damage”	Relationship with arthritis and cartilage damage	Q5	Is this a form of arthritis or cartilage damage?
October 2025	“patellofemoral pain syndrome primary treatment”	First-line treatment	Q6	What is the primary treatment for PFPS?
October 2025	“patellofemoral pain syndrome recovery time prognosis”	Expected duration and recovery	Q7	How long will it take for my knee to heal?
October 2025	“does patellofemoral pain syndrome require surgery”	Surgical indications	Q8	Do I need surgery?
October 2025	“physical therapy for patellofemoral pain syndrome”	Role of physical therapy	Q9	Do I need physical therapy?
October 2025	“conservative treatment options for patellofemoral pain syndrome”	Nonoperative treatment options other than physical therapy	Q10	Are there any conservative options besides physical therapy?
October 2025	“patellofemoral pain syndrome rest or exercise”	Activity modification and continued exercise	Q11	Should I rest completely or keep exercising?
October 2025	“immediate pain relief for patellofemoral pain syndrome”	Short-term symptom relief	Q12	What can I do for immediate pain relief?
October 2025	“knee brace taping for patellofemoral pain syndrome”	Bracing and patellar taping	Q13	Will a knee brace or taping help?
October 2025	“patellofemoral pain syndrome cracking grinding crepitus”	Crepitus and joint sounds	Q14	Why do I hear cracking or grinding (crepitus)?
October 2025	“return to sport running after patellofemoral pain syndrome”	Return to sport and running	Q15	When can I return to my sport or running?
October 2025	“patellofemoral pain syndrome recurrence risk”	Risk of recurrent symptoms	Q16	What is the chance of this pain coming back?
October 2025	“how to prevent patellofemoral pain syndrome recurrence”	Primary and secondary prevention	Q17	How can I prevent PFPS from happening again?

Searches were conducted in English using Google in October 2025. Questions addressing the definition, causes, symptoms, treatment, prognosis, physical activity, return to sport, recurrence, and prevention of PFPS were considered eligible. Duplicate questions and semantically overlapping formulations were merged by consensus among four orthopedic surgeons. Questions requiring interpretation of patient-specific examination findings, medical records, or imaging studies were excluded. The selected questions were revised into lay language without changing their intended clinical meaning.

Supplementary Table S2. Access details and query settings of the evaluated large language models.

Developer	Model	Platform and Access Type	User-Facing Response Mode/Reasoning Setting	Access Date
OpenAI	GPT-5	ChatGPT web interface, paid subscription	Standard default interface; no optional thinking or extended-reasoning mode was manually activated	20 October 2025
Google	Gemini 2.5 Pro	Gemini web interface, paid subscription	Standard default interface; no optional thinking or extended-reasoning mode was manually activated	20 October 2025
xAI	Grok 4	Grok web interface, paid SuperGrok subscription	Standard default interface; no optional thinking or extended-reasoning mode was manually activated	20 October 2025
DeepSeek	DeepSeek-V3.2-Exp	DeepSeek web interface, paid account access	Fast mode; reasoning mode was not activated	20 October 2025

A separate chat session was opened for each model, and default user-facing settings were used; no optional thinking or extended-reasoning mode was manually activated, and DeepSeek-V3.2-Exp was queried using its Fast mode. The same standardized patient scenario and the complete set of 17 questions were submitted together to each model in a single prompt using identical wording and question order. Only the initial combined response generated by each model was retained. No follow-up prompts, regeneration requests, clarification prompts, or manual modifications were applied. No patient-specific or identifiable health information was entered. All four models were accessed and queried on 20 October 2025.

Supplementary Table S3. Characteristics of the evaluators.

Evaluator	Age at Evaluation, Years	Sex	Orthopedic Training and Practice, Years	Specialist Experience, Years	Experience in Managing PFPS	Previous LLM Use
Evaluator 1	29–30	Male	7	2	Routine clinical management	Regular
Evaluator 2	35–36	Male	12	7	Routine clinical management	Regular
Evaluator 3	32–33	Male	9	4	Routine clinical management	Regular
Evaluator 4	33–34	Male	9	4	Routine clinical management	Regular

Orthopedic training and practice includes five years of residency training and subsequent specialist practice. All evaluators were orthopedic surgeons, members of the research team, and authors of the manuscript. Each evaluator had clinical experience in the assessment and nonoperative management of anterior knee pain and PFPS. Their characteristics are presented descriptively to provide context for the DISCERN assessments and were not included as variables in the statistical analyses.

COMBINED SUPPLEMENTARY MATERIAL - PART S1

Complete Large Language Model Responses and DISCERN Evaluation Framework

Supplementary material for the manuscript

Evaluation and comparison of large language model responses to frequently asked questions regarding patellofemoral pain syndrome: a quality and readability assessment study

Oktay Polat, Berk Koncalıoğlu, Mert Gündoğdu, and Emrecan Akgün

Document note. This combined supplementary material contains two parts. Part S1 includes the standardized prompt, question mapping, DISCERN evaluation framework, and the complete responses generated by all four LLMs for all 17 questions. Part S2 includes all 272 anonymized DISCERN scores used in the analyses. No response content or source scores were modified; only presentation formatting was standardized.

1. Standardized Prompt and Question Set

The following scenario and 17 questions were submitted to each model in a single prompt using identical wording and question order.

> I went to the orthopaedic outpatient clinic because of knee pain. I was told that I have patellofemoral pain syndrome (PFPS). I have a few questions regarding this condition. What exactly is PFPS? What causes my pain? Is this the same as "Runner's Knee" or PFS? What activities make the pain worse? Is this a form of arthritis or cartilage damage? What is the primary treatment for PFPS? How long will it take for my knee to heal? Do I need surgery? Do I need physical therapy ? Are there any conservative options besides physical therapy ? Should I rest completely or keep exercising? What can I do for immediate pain relief? Will a knee brace or taping help? Why do I hear cracking or grinding (crepitus)? When can I return to my sport or running? What is the chance of this pain coming back? How can I prevent PFPS from happening again?

2. Question Mapping

Question codes used in the manuscript and scoring dataset are listed below.

Code	Final question
Q1	What exactly is patellofemoral pain syndrome?
Q2	What causes my knee pain?
Q3	Is this the same as “runner’s knee” or patellofemoral syndrome?
Q4	What activities make the pain worse?
Q5	Is this a form of arthritis or cartilage damage?
Q6	What is the primary treatment for PFPS?
Q7	How long will it take for my knee to heal?
Q8	Do I need surgery?
Q9	Do I need physical therapy?
Q10	Are there any conservative options besides physical therapy?
Q11	Should I rest completely or keep exercising?
Q12	What can I do for immediate pain relief?
Q13	Will a knee brace or taping help?
Q14	Why do I hear cracking or grinding (crepitus)?
Q15	When can I return to my sport or running?
Q16	What is the chance of this pain coming back?
Q17	How can I prevent PFPS from happening again?

3. DISCERN Evaluation Framework

Each of the 68 question-specific answer segments was independently evaluated by four orthopaedic surgeons using the 16-item DISCERN instrument. Model identifiers were removed, the answer segments were randomized, and each evaluator scored the responses independently.

Each DISCERN item was rated on a five-point scale. A score of 1 indicated that the criterion was not met, scores of 2-4 indicated that it was met to varying degrees, and a score of 5 indicated that it was fully met. The 16 item scores were summed to produce a total score ranging from 16 to 80. No categorical quality bands were imposed in the present analysis.

The criteria and brief scoring guidance used to support consistent interpretation are summarized below.

Item	DISCERN criterion	Brief scoring guidance
1	Are the aims clear?	The purpose of the response is explicitly stated.
2	Does it achieve its aims?	The content addresses the stated purpose and patient question.
3	Is it relevant?	The information is relevant to PFPS and to the intended patient audience.
4	Are sources of information clear?	Key information sources or references are identified where appropriate.
5	Is the information up to date?	The response indicates the currency or timing of the information where relevant.
6	Is it balanced and unbiased?	Benefits, limitations, and drawbacks are presented fairly.
7	Are additional sources of support or information provided?	The response points readers toward appropriate further help or information.
8	Does it discuss areas of uncertainty?	Limitations of evidence and areas of uncertainty are acknowledged.
9	Does it describe how each treatment works?	The mechanism or intended approach of each treatment option is explained.
10	Does it describe the benefits of each treatment?	Expected outcomes or potential benefits are described.
11	Does it describe the risks of each treatment?	Potential adverse effects, limitations, or risks are described.
12	Does it describe what happens with no treatment?	Possible outcomes of no treatment, delayed treatment, or watchful waiting are addressed.
13	Does it describe impacts on quality of life?	Effects on daily activities, function, and quality of life are considered.
14	Is it clear that there may be more than one treatment choice?	Multiple reasonable treatment or management options are presented.
15	Does it support shared decision-making?	The response helps readers compare options and participate in decisions.
16	Overall quality rating	Overall judgment of the response considering the preceding 15 items.

Scoring note. The brief guidance above was used as a practical aid for consistent interpretation and did not modify the wording or five-point structure of the DISCERN instrument.

4. Complete Initial Model Responses

Each model was queried in a separate chat session. The responses below are presented in the model order used throughout the manuscript.

4.1 GPT-5

**Developer**	OpenAI
**Model**	GPT-5
**Access date**	20 October 2025
**Response mode**	Standard default user-facing interface; no optional thinking or extended-reasoning mode was manually activated

Complete initial response

Those are excellent and very common questions about Patellofemoral Pain Syndrome (PFPS). Let’s go through them carefully, one by one.

What Is PFPS?

Patellofemoral Pain Syndrome (PFPS) refers to pain around or behind the kneecap (patella), where it meets the thigh bone (femur). The pain typically comes from irritation of the soft tissues, cartilage, or the joint surfaces between the patella and femur.

What Causes the Pain?

PFPS is often due to:

- Overuse or repetitive knee motion, especially bending (e.g. running, squatting, climbing stairs)
- Poor alignment or tracking of the kneecap in its groove
- Muscle imbalance or weakness, particularly of the quadriceps (especially the vastus medialis oblique) and hip stabilizers
- Tight structures such as the iliotibial (IT) band or hamstrings
- Sudden increase in activity (mileage, intensity, or hill running)

The pain isn’t typically from a single injury — it’s usually mechanical irritation and soft-tissue stress.

Is It the Same as “Runner’s Knee” or “PFS”?

Yes — “Runner’s Knee” and Patellofemoral Syndrome (PFS) are informal names for PFPS. It’s one of the most common knee problems in runners and athletes.

What Activities Make It Worse?

Pain often increases with:

- Running (especially downhill)
- Climbing or descending stairs
- Squatting or kneeling
- Sitting for long periods (“theater sign”)
- Jumping sports

Is This Arthritis or Cartilage Damage?

Usually not. PFPS is a soft-tissue and mechanical pain syndrome, not structural cartilage loss. However, chronic untreated PFPS may eventually lead to cartilage wear (chondromalacia patellae), but that’s less common.

Primary Treatment

The mainstay of treatment is conservative:

- Activity modification
- Physical therapy — especially strengthening and flexibility of hip and quadriceps muscles
- Stretching tight structures (IT band, hamstrings)
- Taping or bracing for temporary relief

Healing Time

Most people improve significantly in 6–12 weeks with proper management. Some may need up to 3–6 months for full recovery, depending on chronicity and activity level.

Is Surgery Needed?

Almost never. Surgery is reserved for rare cases where there is clear structural misalignment or cartilage injury that hasn’t improved after 6+ months of dedicated rehabilitation.

Do I Need Physical Therapy?

Yes, physical therapy is highly recommended. It’s the single most effective intervention. A PT can teach:

- Strengthening of the hip abductors, glutes, and quadriceps
- Taping or patellar tracking correction
- Gait and running form adjustments

Conservative Options Besides PT

- Activity modification (avoiding painful motions)
- Nonsteroidal anti-inflammatory drugs (NSAIDs) short-term
- Ice therapy
- Knee brace or taping
- Foot orthotics if you have flat feet or overpronation

Rest or Keep Exercising?

Avoid painful activities, but don't rest completely. Continue low-impact exercise:

- Cycling (with low resistance)
- Swimming
- Walking (if pain-free)

Total rest leads to muscle weakness, which can worsen the problem.

Immediate Pain Relief

- Ice (15–20 min after activity)
- NSAIDs (ibuprofen or naproxen, if safe for you)
- Knee taping or sleeve brace
- Avoid deep squats or stairs temporarily

Knee Brace or Taping

Yes — patellar taping (McConnell taping) or a patellofemoral tracking brace may relieve pain short-term by improving patellar alignment.

However, it should complement therapy, not replace it.

Cracking or Grinding (Crepitus)

The crackling sound is common and usually harmless. It comes from air bubbles or movement of soft tissue over the joint — not necessarily cartilage damage. As long as it's not painful, it's not concerning.

Return to Sport / Running

You can resume gradually when:

- Pain-free during daily activities
- Able to do squats, stairs, and jogging without pain
- Quadriceps and hip strength are symmetrical

Return should be gradual over weeks, not all at once.

Recurrence Risk

Recurrence is possible if:

- Muscle imbalances return
- You suddenly increase activity
- You don't maintain flexibility and strength

Regular conditioning and proper biomechanics reduce recurrence risk substantially.

Prevention

- Warm up and stretch before exercise
- Strengthen hip and thigh muscles
- Gradually increase training load
- Use proper footwear
- Maintain healthy weight
- Avoid running on hard or uneven surfaces

Would you like me to create a simple exercise plan (with images or descriptions) for PFPS rehabilitation that you can follow at home? It can cover stretching, strengthening, and progression to sport safely.

4.2 Gemini 2.5 Pro

**Developer**	Google
**Model**	Gemini 2.5 Pro
**Access date**	20 October 2025
**Response mode**	Standard default user-facing interface; no optional thinking or extended-reasoning mode was manually activated

Complete initial response

Understanding Patellofemoral Pain Syndrome (PFPS): A Comprehensive Guide

For individuals experiencing anterior knee pain, a diagnosis of Patellofemoral Pain Syndrome (PFPS) is common. This guide addresses frequent questions about the condition, offering clarity on its causes, treatment, and prevention.

What Exactly is PFPS and What Causes My Pain?

Patellofemoral Pain Syndrome is characterized by pain in the front of the knee, around or behind the kneecap (patella). The discomfort arises from stress and irritation of the cartilage on the underside of the patella and the groove in the thighbone (femur) in which it glides. The pain is often a result of improper tracking of the kneecap, which can be caused by a variety of factors, including:

Overuse: Repetitive activities that place stress on the knee joint, such as running, squatting, and climbing stairs.

Muscle Imbalances: Weakness in the quadriceps (thigh muscles) and hip abductor muscles can lead to poor alignment of the kneecap. Tightness in the hamstrings and iliotibial (IT) band can also contribute.

Anatomical Factors: The natural alignment of an individual's legs, the shape of the kneecap, and flat feet can predispose them to PFPS.

Is This the Same as "Runner's Knee" or PFS?

Yes, Patellofemoral Pain Syndrome is often colloquially referred to as "Runner's Knee" due to its high prevalence among runners. The abbreviation PFS is also commonly used to denote the same condition.

What Activities Make the Pain Worse?

Activities that increase the pressure between the kneecap and the thighbone typically exacerbate the pain. These include:

- Running, especially downhill
- Going up and down stairs
- Squatting or kneeling
- Prolonged sitting with the knees bent (sometimes called "moviegoer's knee")
- Jumping

Is This a Form of Arthritis or Cartilage Damage?

While PFPS involves irritation of the cartilage, it is not in itself a form of arthritis in its early stages. However, if left unmanaged, the chronic irritation and improper tracking can contribute to the wearing down of the articular cartilage, potentially leading to patellofemoral osteoarthritis over time.

What is the Primary Treatment for PFPS and Do I Need Physical Therapy?

The cornerstone of treatment for PFPS is conservative and multifaceted, with physical therapy playing a crucial role. A physical therapist will design a program tailored to your specific needs, which will likely include:

Strengthening Exercises: Focusing on the quadriceps (particularly the vastus medialis obliquus or VMO), hip abductors, and core muscles to improve kneecap tracking and pelvic stability.

Stretching: Addressing tightness in the hamstrings, quadriceps, iliotibial band, and calf muscles.

Activity Modification: Guidance on how to alter your activities to reduce stress on the knee.

Gait and Movement Analysis: Assessing and correcting your walking and running mechanics.

Are There Any Conservative Options Besides Physical Therapy?

Yes, other conservative treatments can be used in conjunction with physical therapy:

Activity Modification and Relative Rest: Reducing or temporarily stopping activities that cause pain is essential. Complete rest is generally not recommended, but a period of reduced activity allows the irritation to subside.

Ice: Applying ice packs to the front of the knee for 15-20 minutes after activity can help reduce pain and inflammation.

Pain Relief Medications: Over-the-counter nonsteroidal anti-inflammatory drugs (NSAIDs) like ibuprofen may be recommended to manage pain and inflammation.

How Long Will It Take for My Knee to Heal and Do I Need Surgery?

The healing time for PFPS varies depending on the severity of the condition and adherence to the treatment plan. It can take anywhere from a few weeks to several months to achieve significant pain relief and return to full activity. Surgery is rarely necessary for PFPS and is only considered in persistent cases where conservative treatments have failed to provide relief.

Should I Rest Completely or Keep Exercising?

A balance is key. While you should avoid activities that cause significant pain, complete rest can lead to muscle weakness, which can worsen the problem. The goal is to find a level of activity that does not aggravate your symptoms while you work on the underlying causes through your physical therapy program. Low-impact activities like swimming or using an elliptical trainer may be good alternatives.

What Can I Do for Immediate Pain Relief? Will a Knee Brace or Taping Help?

For immediate relief, resting the knee and applying ice can be effective. A simple knee sleeve or brace can sometimes provide support and help with pain. Patellar taping, often done by a physical therapist, can help to correct the tracking of the kneecap and provide significant short-term pain relief, making it easier to perform strengthening exercises.

Why Do I Hear Cracking or Grinding (Crepitus)?

The cracking or grinding sound, known as crepitus, is common with PFPS. It is often caused by the roughened cartilage on the back of the patella moving against the femur. While it can be unsettling, it is not always a sign of significant damage, especially if it is not accompanied by pain.

When Can I Return to My Sport or Running?

Returning to your sport or running should be a gradual process guided by your pain levels and the advice of your doctor or physical therapist. A general guideline is to be pain-free with daily activities and to have regained sufficient strength and flexibility. A graded return to activity, starting with shorter durations and lower intensity, is crucial to prevent re-injury.

What is the Chance of This Pain Coming Back and How Can I Prevent It?

PFPS has a tendency to recur if the underlying causes are not addressed. The best prevention strategy is to continue with the strengthening and flexibility exercises prescribed by your physical therapist even after your pain has resolved. Other preventative measures include:

Proper Footwear: Wearing supportive shoes that are appropriate for your foot type and activity.

Gradual Progression of Activity: Avoiding sudden increases in the intensity or duration of your exercise.

Maintaining a Healthy Weight: Reducing excess body weight can significantly decrease the stress on your knees.

Proper Warm-up and Cool-down: Always preparing your muscles for activity and stretching afterward.

By understanding the nature of PFPS and actively participating in a comprehensive treatment and prevention plan, you can effectively manage your knee pain and return to the activities you enjoy.

4.3 Grok 4

**Developer**	xAI
**Model**	Grok 4
**Access date**	20 October 2025
**Response mode**	Standard default user-facing interface; no optional thinking or extended-reasoning mode was manually activated

Complete initial response

What exactly is PFPS?

Patellofemoral pain syndrome (PFPS) is a condition characterized by pain in the front of the knee, around or under the kneecap (patella). It occurs when the patella doesn't move smoothly in its groove on the femur (thigh bone), leading to irritation of the surrounding soft tissues, such as tendons, synovial tissue, or the fat pad beneath the patella. In some cases, it may involve chondromalacia patella, which is softening of the cartilage under the kneecap, but the pain primarily comes from inflammation rather than the cartilage itself.

What causes my pain?

The exact cause isn't always clear, but common contributors include overuse from repetitive activities like running or jumping, muscle imbalances or weaknesses (especially in the quadriceps, hips, or hamstrings), patellar malalignment where the kneecap doesn't track properly in the femoral groove, sudden increases in activity level, tight muscles or tendons (like hamstrings or Achilles), and sometimes structural factors like a high-riding patella or leg alignment issues. Trauma, surgery, or even changes in footwear can also play a role.

Is this the same as "Runner's Knee" or PFS?

Yes, PFPS is commonly known as "runner's knee" or "jumper's knee," especially since it's prevalent in athletes involved in running and jumping sports. PFS likely refers to patellofemoral syndrome, which is essentially the same condition.

What activities make the pain worse?

Activities that involve repeated knee bending under load, such as running, jumping, squatting, climbing or descending stairs, kneeling, or sitting with bent knees for prolonged periods (like in a car or theater). Changes in training intensity, surfaces, or shoes can also exacerbate it.

Is this a form of arthritis or cartilage damage?

PFPS is not a form of arthritis; it's a pain syndrome related to soft tissue irritation and patellar tracking issues, more common in younger people, whereas arthritis is typically degenerative and affects older adults. It can sometimes involve cartilage softening (chondromalacia patella), but this isn't always present, and the pain isn't directly from cartilage damage since cartilage has no nerves—it's from resulting inflammation.

What is the primary treatment for PFPS?

The primary treatment is conservative and nonsurgical, focusing on relieving pain and addressing underlying causes through rest, ice, physical therapy to strengthen muscles (especially quadriceps and hips), and activity modification.

How long will it take for my knee to heal?

Recovery time varies but most people see improvement in 1-2 months with proper treatment, depending on individual factors like strength, activity level, and adherence to therapy. It can take longer if not managed well.

Do I need surgery?

Surgery is rarely needed and only considered for severe cases that don't respond to conservative treatments. Options might include arthroscopy to remove damaged tissue or realign the patella.

Do I need physical therapy?

Yes, physical therapy is often recommended as a key part of treatment to improve muscle strength, flexibility, and patellar tracking.

Are there any conservative options besides physical therapy?

Yes, options include the RICE method (rest, ice, compression, elevation), over-the-counter pain relievers like NSAIDs (e.g., ibuprofen), activity modification to avoid aggravating movements, weight loss if applicable, orthotics or shoe inserts, and sometimes knee braces for support.

Should I rest completely or keep exercising?

Avoid complete rest; instead, opt for relative rest by stopping aggravating activities while continuing low-impact exercises and strengthening through physical therapy. Switch to activities like swimming or cycling.

What can I do for immediate pain relief?

Apply the RICE method: rest the knee, ice it for 20 minutes several times a day, use compression with a bandage, and elevate it. Take over-the-counter NSAIDs for pain and swelling.

Will a knee brace or taping help?

Knee braces can provide stability and pain relief, especially during early recovery or activity. Taping (like patellar taping) isn't always mentioned but can help with alignment in some cases; consult a professional for proper application.

Why do I hear cracking or grinding (crepitus)?

Crepitus is common in PFPS and results from irregular patellar tracking over the femur, causing popping or cracking sounds. It's usually harmless but can indicate inflammation.

When can I return to my sport or running?

Return gradually once pain-free, after completing physical therapy and strengthening muscles. This typically follows 1-2 months of treatment, but follow your doctor's or therapist's guidance.

What is the chance of this pain coming back?

There's a risk of recurrence if underlying issues like muscle weaknesses or training errors aren't addressed, but proper prevention reduces this.

How can I prevent PFPS from happening again?

Prevent by strengthening leg and hip muscles, warming up and stretching before activity, gradually increasing training intensity, wearing proper footwear, maintaining a healthy weight, and using correct techniques in sports.

4.4 DeepSeek-V3.2-Exp

**Developer**	DeepSeek
**Model**	DeepSeek-V3.2-Exp
**Access date**	20 October 2025
**Response mode**	Fast mode; reasoning mode was not activated

Complete initial response

Of course. It's completely normal to have many questions after a diagnosis. Let's break down your patellofemoral pain syndrome (PFPS) step-by-step. This information will help you understand your condition and actively participate in your recovery.

Disclaimer: This is for informational purposes and does not replace professional medical advice. Always follow the specific guidance of your orthopaedic doctor or physical therapist.

1. What exactly is PFPS?

Patellofemoral Pain Syndrome (PFPS) is a broad term for pain arising from the joint between your kneecap (patella) and the thigh bone (femur). Think of your kneecap as a train on a track (the femoral groove). PFPS occurs when the kneecap doesn't move or align properly on this track, leading to irritation and pain in the surrounding tissues and the bone underneath.

2. What causes my pain?

The pain is primarily caused by excessive pressure or abnormal tracking of the kneecap, which irritates the sensitive structures behind it. Common contributing factors include:

Muscle Imbalances: Weakness in the hip and core muscles (glutes) or the quadriceps (thigh muscles) can cause the knee to collapse inwards, pulling the kneecap out of alignment.

Overuse: A sudden increase in activities that load the knee joint, like running, jumping, or climbing stairs.

Biomechanics: Your individual anatomy, foot mechanics (like flat feet), or gait can contribute.

Direct Trauma: A fall or a blow to the kneecap.

3. Is this the same as "Runner's Knee" or PFS?

Runner's Knee: Yes, this is essentially the same thing. "Runner's Knee" is a common layman's term for PFPS because it's so prevalent in running sports.

PFS (Patellofemoral Syndrome): This is just a different abbreviation for the same condition. PFPS and PFS are used interchangeably.

4. What activities make the pain worse?

Any activity that increases pressure between the kneecap and femur will typically aggravate the pain:

- Walking up or down stairs
- Squatting or lunging
- Running, especially downhill
- Prolonged sitting with bent knees (the "movie sign" or "theater sign")
- Kneeling

5. Is this a form of arthritis or cartilage damage?

Arthritis: No. PFPS is primarily a soft tissue and alignment issue, not a degenerative joint disease like osteoarthritis.

Cartilage Damage: Not initially. PFPS involves irritation and softening of the cartilage on the back of the kneecap (a condition called chondromalacia patellae). However, if left unaddressed for a long time, ongoing irritation can progress to cartilage damage. Think of PFPS as an early warning sign to correct the issue before it causes lasting damage.

6. What is the primary treatment for PFPS?

The primary and most effective treatment is conservative management, with a strong focus on physical therapy. The goal is to correct the underlying muscle imbalances and biomechanics.

7. How long will it take for my knee to heal?

Recovery is highly individual, but with consistent effort, you can expect significant improvement within 4 to 8 weeks. Full recovery, especially for a return to high-level sports, can take 3 months or longer. Patience and consistency are key.

8. Do I need surgery?

Surgery is very rarely needed for PFPS. Over 90% of cases resolve successfully with conservative treatment like physical therapy. Surgery is only considered after many months of diligent, non-surgical treatment has failed.

9. Do I need physical therapy?

Yes, absolutely. Physical therapy is the cornerstone of PFPS treatment. A physical therapist will:

- Assess your specific muscle weaknesses and imbalances.

- Design a personalized exercise program focusing on strengthening your hips, glutes, and quadriceps.
- Teach you proper movement patterns for daily activities and sports.
- Use techniques like taping or manual therapy for immediate pain relief.

10. Are there any conservative options besides physical therapy?

Yes, PT is the main one, but it's often combined with:

Activity Modification: Temporarily reducing or changing painful activities.

Ice: Applying ice after activity for 15-20 minutes to reduce pain and inflammation.

Anti-inflammatory medication (NSAIDs): Like ibuprofen, as advised by your doctor for short-term pain relief.

Foot Orthotics: If you have flat feet, custom or off-the-shelf shoe inserts can help improve leg alignment.

11. Should I rest completely or keep exercising?

Relative rest, not complete rest.

- Stop activities that cause pain (e.g., running, jumping).
- Continue with pain-free cross-training to maintain fitness, such as swimming, cycling (with a high seat), or using an elliptical machine. This "load management" is crucial.

12. What can I do for immediate pain relief?

Ice Therapy: Apply an ice pack to the painful area for 15-20 minutes.

Rest: Get off your feet and avoid aggravating movements.

Elevate: Prop your leg up.

Over-the-counter pain relievers: As recommended by your doctor or pharmacist.

13. Will a knee brace or taping help?

Taping (e.g., McConnell Taping): Can be very helpful, especially in the early stages. It physically pulls the kneecap into a better position, providing immediate pain relief and allowing you to perform strengthening exercises more comfortably. Your physical therapist can teach you how.

Knee Brace/Sleeve: A simple patellar strap or knee sleeve can provide proprioceptive feedback (making you more aware of your knee position) and a sense of support, which many people find reassuring. It doesn't fix the root cause but can be a useful tool.

14. Why do I hear cracking or grinding (crepitus)?

This sound, called crepitus, is common in PFPS. It's often due to the irritated and slightly softened cartilage on the back of the kneecap rubbing on the femur. If it's not painful, it's usually not a cause for concern. Painful grinding, however, should be monitored by your doctor.

15. When can I return to my sport or running?

This is a gradual process. You can return when:

- You have minimal to no pain during daily activities.
- Your strength and flexibility have been restored.
- You can perform sport-specific movements (like hopping or squatting) without pain.
- Your physical therapist will guide you through a phased return-to-sport program.

16. What is the chance of this pain coming back?

PFPS has a high recurrence rate if the underlying causes (weak hips, poor movement patterns) are not fully addressed. This is why the strengthening and education part of physical therapy is so critical for long-term success.

17. How can I prevent PFPS from happening again?

- Maintain Strength: Continue the hip, glute, and core exercises you learn in PT as part of your regular routine.
- Warm Up Properly: Always warm up before exercise.
- Increase Activity Gradually: Follow the 10% rule—don't increase your running mileage or workout intensity by more than 10% per week.
- Wear Appropriate Footwear.
- Listen to Your Body: Don't ignore the early signs of pain.

You've taken a great first step by seeking information. Now, the most important step is to commit to a physical therapy program. It's the most effective way to get you back to a pain-free, active life. Good luck

End of Supplementary File S1

PART S2

Anonymized DISCERN Scoring Dataset

for the manuscript

Evaluation and comparison of large language model responses to frequently asked questions regarding patellofemoral pain syndrome: a quality and readability assessment study

Oktay Polat, Berk Koncalıoğlu, Mert Gündoğdu, and Emrecan Akgün

Document note. This file contains the anonymized total DISCERN scores used in the primary and sensitivity analyses. Each score represents the total score assigned by one evaluator to one question-specific answer segment. Model identities are shown for analytical reproducibility, whereas evaluator identities remain anonymized. No scores were missing, imputed, or manually altered.

1. Dataset Overview

Field	Description
Purpose	To provide the anonymized total DISCERN scores used in the primary and sensitivity analyses.
Unit of analysis	One question-specific answer segment generated by one large language model.
Dataset size	17 questions × 4 models × 4 evaluators = 272 total DISCERN scores.
Score range	Each total DISCERN score ranges from 16 to 80; higher values indicate better information quality and reliability.
Evaluator coding	The four orthopaedic surgeon evaluators are anonymized as Evaluator 1, Evaluator 2, Evaluator 3, and Evaluator 4.
Models	OpenAI GPT-5; Google Gemini 2.5 Pro; xAI Grok 4; DeepSeek-V3.2-Exp.
Primary analysis	For each question-specific answer segment, the mean of the four evaluator scores was calculated.
Sensitivity analysis	For each question-specific answer segment, the median of the four evaluator scores was calculated.
Data integrity	No scores are missing. No imputation or manual modification of the source scores was performed.

2. DISCERN Evaluation Framework

Each of the 68 question-specific answer segments was independently evaluated by four orthopaedic surgeons using the 16-item DISCERN instrument. Model identifiers were removed, the answer segments were randomized, and each evaluator scored the responses independently.

Each DISCERN item was rated on a five-point scale. A score of 1 indicated that the criterion was not met, scores of 2–4 indicated that it was met to varying degrees, and a score of 5 indicated that it was fully met. The 16 item scores were summed to produce a total score ranging from 16 to 80. No categorical quality bands were imposed in the present analysis.

Item	DISCERN criterion	Brief scoring guidance
1	Are the aims clear?	The purpose of the response is explicitly stated.
2	Does it achieve its aims?	The content addresses the stated purpose and patient question.
3	Is it relevant?	The information is relevant to PFPS and to the intended patient audience.
4	Are sources of information clear?	Key information sources or references are identified where appropriate.
5	Is the information up to date?	The response indicates the currency or timing of the information where relevant.
6	Is it balanced and unbiased?	Benefits, limitations, and drawbacks are presented fairly.

Item	DISCERN criterion	Brief scoring guidance
7	Are additional sources of support or information provided?	The response points readers toward appropriate further help or information.
8	Does it discuss areas of uncertainty?	Limitations of evidence and areas of uncertainty are acknowledged.
9	Does it describe how each treatment works?	The mechanism or intended approach of each treatment option is explained.
10	Does it describe the benefits of each treatment?	Expected outcomes or potential benefits are described.
11	Does it describe the risks of each treatment?	Potential adverse effects, limitations, or risks are described.
12	Does it describe what happens with no treatment?	Possible outcomes of no treatment, delayed treatment, or watchful waiting are addressed.
13	Does it describe impacts on quality of life?	Effects on daily activities, function, and quality of life are considered.
14	Is it clear that there may be more than one treatment choice?	Multiple reasonable treatment or management options are presented.
15	Does it support shared decision-making?	The response helps readers compare options and participate in decisions.
16	Overall quality rating	Overall judgment of the response considering the preceding 15 items.

Scoring note. The brief guidance above was used as a practical aid for consistent interpretation and did not modify the wording or five-point structure of the DISCERN instrument.

3. Wide-Format Scoring Dataset

One row is presented for each question–model combination. Evaluator 1–4 columns contain the four independent total DISCERN scores. Mean and median values correspond to the summaries used in the primary and sensitivity analyses, respectively.

Question code	Question	Model	Evaluator 1	Evaluator 2	Evaluator 3	Evaluator 4	Mean score	Median score
Q1	What exactly is patellofemoral pain syndrome?	GPT-5	49	48	42	34	43.25	45
Q1	What exactly is patellofemoral pain syndrome?	Gemini 2.5 Pro	54	64	47	41	51.5	50.5
Q1	What exactly is patellofemoral pain syndrome?	Grok 4	57	56	48	49	52.5	52.5
Q1	What exactly is patellofemoral pain syndrome?	DeepSeek-V3.2-Exp	45	54	46	36	45.25	45.5
Q2	What causes my knee pain?	GPT-5	55	62	47	36	50	51
Q2	What causes my knee pain?	Gemini 2.5 Pro	51	56	49	41	49.25	50
Q2	What causes my knee pain?	Grok 4	59	64	52	49	56	55.5
Q2	What causes my knee pain?	DeepSeek-V3.2-Exp	47	65	49	36	49.25	48
Q3	Is this the same as "runner’s knee" or patellofemoral syndrome?	GPT-5	44	58	47	34	45.75	45.5
Q3	Is this the same as "runner’s knee" or patellofemoral syndrome?	Gemini 2.5 Pro	40	64	50	39	48.25	45
Q3	Is this the same as "runner’s knee" or patellofemoral syndrome?	Grok 4	46	64	51	46	51.75	48.5
Q3	Is this the same as "runner’s knee" or patellofemoral syndrome?	DeepSeek-V3.2-Exp	42	60	47	36	46.25	44.5
Q4	What activities make the pain worse?	GPT-5	46	64	47	37	48.5	46.5
Q4	What activities make the pain worse?	Gemini 2.5 Pro	45	64	51	41	50.25	48
Q4	What activities make the pain worse?	Grok 4	52	64	53	46	53.75	52.5
Q4	What activities make the pain worse?	DeepSeek-V3.2-Exp	47	64	48	40	49.75	47.5
Q5	Is this a form of arthritis or cartilage damage?	GPT-5	57	60	49	34	50	53
Q5	Is this a form of arthritis or cartilage damage?	Gemini 2.5 Pro	49	64	53	36	50.5	51
Q5	Is this a form of arthritis or cartilage damage?	Grok 4	59	77	55	44	58.75	57
Q5	Is this a form of arthritis or cartilage damage?	DeepSeek-V3.2-Exp	63	70	50	36	54.75	56.5
Q6	What is the primary treatment for PFPS?	GPT-5	64	56	56	39	53.75	56
Q6	What is the primary treatment for PFPS?	Gemini 2.5 Pro	66	66	55	42	57.25	60.5

Question code	Question	Model	Evaluator 1	Evaluator 2	Evaluator 3	Evaluator 4	Mean score	Median score
Q6	What is the primary treatment for PFPS?	Grok 4	63	56	62	45	56.5	59
Q6	What is the primary treatment for PFPS?	DeepSeek-V3.2-Exp	52	61	53	38	51	52.5
Q7	How long will it take for my knee to heal?	GPT-5	57	62	48	34	50.25	52.5
Q7	How long will it take for my knee to heal?	Gemini 2.5 Pro	66	60	52	43	55.25	56
Q7	How long will it take for my knee to heal?	Grok 4	62	56	54	45	54.25	55
Q7	How long will it take for my knee to heal?	DeepSeek-V3.2-Exp	57	62	51	36	51.5	54
Q8	Do I need surgery?	GPT-5	62	67	48	34	52.75	55
Q8	Do I need surgery?	Gemini 2.5 Pro	64	62	53	41	55	57.5
Q8	Do I need surgery?	Grok 4	62	69	54	45	57.5	58
Q8	Do I need surgery?	DeepSeek-V3.2-Exp	64	60	51	36	52.75	55.5
Q9	Do I need physical therapy?	GPT-5	64	65	56	39	56	60
Q9	Do I need physical therapy?	Gemini 2.5 Pro	68	68	55	39	57.5	61.5
Q9	Do I need physical therapy?	Grok 4	54	60	62	42	54.5	57
Q9	Do I need physical therapy?	DeepSeek-V3.2-Exp	72	67	53	35	56.75	60
Q10	Are there any conservative options besides physical therapy?	GPT-5	64	60	51	39	53.5	55.5
Q10	Are there any conservative options besides physical therapy?	Gemini 2.5 Pro	63	62	50	39	53.5	56
Q10	Are there any conservative options besides physical therapy?	Grok 4	64	65	51	49	57.25	57.5
Q10	Are there any conservative options besides physical therapy?	DeepSeek-V3.2-Exp	70	70	51	40	57.75	60.5
Q11	Should I rest completely or keep exercising?	GPT-5	70	60	56	39	56.25	58
Q11	Should I rest completely or keep exercising?	Gemini 2.5 Pro	66	64	60	43	58.25	62
Q11	Should I rest completely or keep exercising?	Grok 4	68	64	58	44	58.5	61
Q11	Should I rest completely or keep exercising?	DeepSeek-V3.2-Exp	67	64	59	36	56.5	61.5
Q12	What can I do for immediate pain relief?	GPT-5	66	64	56	39	56.25	60
Q12	What can I do for immediate pain relief?	Gemini 2.5 Pro	68	64	60	37	57.25	62
Q12	What can I do for immediate pain relief?	Grok 4	62	58	61	46	56.75	59.5
Q12	What can I do for immediate pain relief?	DeepSeek-V3.2-Exp	65	67	59	39	57.5	62
Q13	Will a knee brace or taping help?	GPT-5	66	60	56	38	55	58

Question code	Question	Model	Evaluator 1	Evaluator 2	Evaluator 3	Evaluator 4	Mean score	Median score
Q13	Will a knee brace or taping help?	Gemini 2.5 Pro	67	64	55	39	56.25	59.5
Q13	Will a knee brace or taping help?	Grok 4	62	57	57	42	54.5	57
Q13	Will a knee brace or taping help?	DeepSeek-V3.2-Exp	72	64	59	38	58.25	61.5
Q14	Why do I hear cracking or grinding (crepitus)?	GPT-5	55	61	56	36	52	55.5
Q14	Why do I hear cracking or grinding (crepitus)?	Gemini 2.5 Pro	48	64	57	38	51.75	52.5
Q14	Why do I hear cracking or grinding (crepitus)?	Grok 4	52	61	56	45	53.5	54
Q14	Why do I hear cracking or grinding (crepitus)?	DeepSeek-V3.2-Exp	55	67	55	37	53.5	55
Q15	When can I return to my sport or running?	GPT-5	65	60	56	37	54.5	58
Q15	When can I return to my sport or running?	Gemini 2.5 Pro	65	67	57	41	57.5	61
Q15	When can I return to my sport or running?	Grok 4	59	61	56	42	54.5	57.5
Q15	When can I return to my sport or running?	DeepSeek-V3.2-Exp	65	72	55	38	57.5	60
Q16	What is the chance of this pain coming back?	GPT-5	63	67	56	38	56	59.5
Q16	What is the chance of this pain coming back?	Gemini 2.5 Pro	73	64	52	42	57.75	58
Q16	What is the chance of this pain coming back?	Grok 4	60	64	53	43	55	56.5
Q16	What is the chance of this pain coming back?	DeepSeek-V3.2-Exp	62	72	57	38	57.25	59.5
Q17	How can I prevent PFPS from happening again?	GPT-5	67	67	51	36	55.25	59
Q17	How can I prevent PFPS from happening again?	Gemini 2.5 Pro	70	62	52	42	56.5	57
Q17	How can I prevent PFPS from happening again?	Grok 4	59	61	54	49	55.75	56.5
Q17	How can I prevent PFPS from happening again?	DeepSeek-V3.2-Exp	71	70	51	40	58	60.5

4. Long-Format Scoring Dataset

The same 272 total DISCERN scores are presented below in long format, with one row per evaluator score. This structure is provided to facilitate reproducible statistical analysis.

Question code	Question	Model	Evaluator	Total DISCERN score
Q1	What exactly is patellofemoral pain syndrome?	GPT-5	Evaluator 1	49
Q1	What exactly is patellofemoral pain syndrome?	GPT-5	Evaluator 2	48
Q1	What exactly is patellofemoral pain syndrome?	GPT-5	Evaluator 3	42
Q1	What exactly is patellofemoral pain syndrome?	GPT-5	Evaluator 4	34
Q1	What exactly is patellofemoral pain syndrome?	Gemini 2.5 Pro	Evaluator 1	54

Question code	Question	Model	Evaluator	Total DISCERN score
Q1	What exactly is patellofemoral pain syndrome?	Gemini 2.5 Pro	Evaluator 2	64
Q1	What exactly is patellofemoral pain syndrome?	Gemini 2.5 Pro	Evaluator 3	47
Q1	What exactly is patellofemoral pain syndrome?	Gemini 2.5 Pro	Evaluator 4	41
Q1	What exactly is patellofemoral pain syndrome?	Grok 4	Evaluator 1	57
Q1	What exactly is patellofemoral pain syndrome?	Grok 4	Evaluator 2	56
Q1	What exactly is patellofemoral pain syndrome?	Grok 4	Evaluator 3	48
Q1	What exactly is patellofemoral pain syndrome?	Grok 4	Evaluator 4	49
Q1	What exactly is patellofemoral pain syndrome?	DeepSeek-V3.2-Exp	Evaluator 1	45
Q1	What exactly is patellofemoral pain syndrome?	DeepSeek-V3.2-Exp	Evaluator 2	54
Q1	What exactly is patellofemoral pain syndrome?	DeepSeek-V3.2-Exp	Evaluator 3	46
Q1	What exactly is patellofemoral pain syndrome?	DeepSeek-V3.2-Exp	Evaluator 4	36
Q2	What causes my knee pain?	GPT-5	Evaluator 1	55
Q2	What causes my knee pain?	GPT-5	Evaluator 2	62
Q2	What causes my knee pain?	GPT-5	Evaluator 3	47
Q2	What causes my knee pain?	GPT-5	Evaluator 4	36
Q2	What causes my knee pain?	Gemini 2.5 Pro	Evaluator 1	51
Q2	What causes my knee pain?	Gemini 2.5 Pro	Evaluator 2	56
Q2	What causes my knee pain?	Gemini 2.5 Pro	Evaluator 3	49
Q2	What causes my knee pain?	Gemini 2.5 Pro	Evaluator 4	41
Q2	What causes my knee pain?	Grok 4	Evaluator 1	59
Q2	What causes my knee pain?	Grok 4	Evaluator 2	64
Q2	What causes my knee pain?	Grok 4	Evaluator 3	52
Q2	What causes my knee pain?	Grok 4	Evaluator 4	49
Q2	What causes my knee pain?	DeepSeek-V3.2-Exp	Evaluator 1	47
Q2	What causes my knee pain?	DeepSeek-V3.2-Exp	Evaluator 2	65
Q2	What causes my knee pain?	DeepSeek-V3.2-Exp	Evaluator 3	49
Q2	What causes my knee pain?	DeepSeek-V3.2-Exp	Evaluator 4	36
Q3	Is this the same as "runner’s knee" or patellofemoral syndrome?	GPT-5	Evaluator 1	44
Q3	Is this the same as "runner’s knee" or patellofemoral syndrome?	GPT-5	Evaluator 2	58
Q3	Is this the same as "runner’s knee" or patellofemoral syndrome?	GPT-5	Evaluator 3	47
Q3	Is this the same as "runner’s knee" or patellofemoral syndrome?	GPT-5	Evaluator 4	34
Q3	Is this the same as "runner’s knee" or patellofemoral syndrome?	Gemini 2.5 Pro	Evaluator 1	40
Q3	Is this the same as "runner’s knee" or patellofemoral syndrome?	Gemini 2.5 Pro	Evaluator 2	64
Q3	Is this the same as "runner’s knee" or patellofemoral syndrome?	Gemini 2.5 Pro	Evaluator 3	50
Q3	Is this the same as "runner’s knee" or patellofemoral syndrome?	Gemini 2.5 Pro	Evaluator 4	39

Question code	Question	Model	Evaluator	Total DISCERN score
Q3	Is this the same as "runner’s knee" or patellofemoral syndrome?	Grok 4	Evaluator 1	46
Q3	Is this the same as "runner’s knee" or patellofemoral syndrome?	Grok 4	Evaluator 2	64
Q3	Is this the same as "runner’s knee" or patellofemoral syndrome?	Grok 4	Evaluator 3	51
Q3	Is this the same as "runner’s knee" or patellofemoral syndrome?	Grok 4	Evaluator 4	46
Q3	Is this the same as "runner’s knee" or patellofemoral syndrome?	DeepSeek-V3.2-Exp	Evaluator 1	42
Q3	Is this the same as "runner’s knee" or patellofemoral syndrome?	DeepSeek-V3.2-Exp	Evaluator 2	60
Q3	Is this the same as "runner’s knee" or patellofemoral syndrome?	DeepSeek-V3.2-Exp	Evaluator 3	47
Q3	Is this the same as "runner’s knee" or patellofemoral syndrome?	DeepSeek-V3.2-Exp	Evaluator 4	36
Q4	What activities make the pain worse?	GPT-5	Evaluator 1	46
Q4	What activities make the pain worse?	GPT-5	Evaluator 2	64
Q4	What activities make the pain worse?	GPT-5	Evaluator 3	47
Q4	What activities make the pain worse?	GPT-5	Evaluator 4	37
Q4	What activities make the pain worse?	Gemini 2.5 Pro	Evaluator 1	45
Q4	What activities make the pain worse?	Gemini 2.5 Pro	Evaluator 2	64
Q4	What activities make the pain worse?	Gemini 2.5 Pro	Evaluator 3	51
Q4	What activities make the pain worse?	Gemini 2.5 Pro	Evaluator 4	41
Q4	What activities make the pain worse?	Grok 4	Evaluator 1	52
Q4	What activities make the pain worse?	Grok 4	Evaluator 2	64
Q4	What activities make the pain worse?	Grok 4	Evaluator 3	53
Q4	What activities make the pain worse?	Grok 4	Evaluator 4	46
Q4	What activities make the pain worse?	DeepSeek-V3.2-Exp	Evaluator 1	47
Q4	What activities make the pain worse?	DeepSeek-V3.2-Exp	Evaluator 2	64
Q4	What activities make the pain worse?	DeepSeek-V3.2-Exp	Evaluator 3	48
Q4	What activities make the pain worse?	DeepSeek-V3.2-Exp	Evaluator 4	40
Q5	Is this a form of arthritis or cartilage damage?	GPT-5	Evaluator 1	57
Q5	Is this a form of arthritis or cartilage damage?	GPT-5	Evaluator 2	60
Q5	Is this a form of arthritis or cartilage damage?	GPT-5	Evaluator 3	49
Q5	Is this a form of arthritis or cartilage damage?	GPT-5	Evaluator 4	34
Q5	Is this a form of arthritis or cartilage damage?	Gemini 2.5 Pro	Evaluator 1	49
Q5	Is this a form of arthritis or cartilage damage?	Gemini 2.5 Pro	Evaluator 2	64
Q5	Is this a form of arthritis or cartilage damage?	Gemini 2.5 Pro	Evaluator 3	53
Q5	Is this a form of arthritis or cartilage damage?	Gemini 2.5 Pro	Evaluator 4	36
Q5	Is this a form of arthritis or cartilage damage?	Grok 4	Evaluator 1	59
Q5	Is this a form of arthritis or cartilage damage?	Grok 4	Evaluator 2	77
Q5	Is this a form of arthritis or cartilage damage?	Grok 4	Evaluator 3	55

Question code	Question	Model	Evaluator	Total DISCERN score
Q5	Is this a form of arthritis or cartilage damage?	Grok 4	Evaluator 4	44
Q5	Is this a form of arthritis or cartilage damage?	DeepSeek-V3.2-Exp	Evaluator 1	63
Q5	Is this a form of arthritis or cartilage damage?	DeepSeek-V3.2-Exp	Evaluator 2	70
Q5	Is this a form of arthritis or cartilage damage?	DeepSeek-V3.2-Exp	Evaluator 3	50
Q5	Is this a form of arthritis or cartilage damage?	DeepSeek-V3.2-Exp	Evaluator 4	36
Q6	What is the primary treatment for PFPS?	GPT-5	Evaluator 1	64
Q6	What is the primary treatment for PFPS?	GPT-5	Evaluator 2	56
Q6	What is the primary treatment for PFPS?	GPT-5	Evaluator 3	56
Q6	What is the primary treatment for PFPS?	GPT-5	Evaluator 4	39
Q6	What is the primary treatment for PFPS?	Gemini 2.5 Pro	Evaluator 1	66
Q6	What is the primary treatment for PFPS?	Gemini 2.5 Pro	Evaluator 2	66
Q6	What is the primary treatment for PFPS?	Gemini 2.5 Pro	Evaluator 3	55
Q6	What is the primary treatment for PFPS?	Gemini 2.5 Pro	Evaluator 4	42
Q6	What is the primary treatment for PFPS?	Grok 4	Evaluator 1	63
Q6	What is the primary treatment for PFPS?	Grok 4	Evaluator 2	56
Q6	What is the primary treatment for PFPS?	Grok 4	Evaluator 3	62
Q6	What is the primary treatment for PFPS?	Grok 4	Evaluator 4	45
Q6	What is the primary treatment for PFPS?	DeepSeek-V3.2-Exp	Evaluator 1	52
Q6	What is the primary treatment for PFPS?	DeepSeek-V3.2-Exp	Evaluator 2	61
Q6	What is the primary treatment for PFPS?	DeepSeek-V3.2-Exp	Evaluator 3	53
Q6	What is the primary treatment for PFPS?	DeepSeek-V3.2-Exp	Evaluator 4	38
Q7	How long will it take for my knee to heal?	GPT-5	Evaluator 1	57
Q7	How long will it take for my knee to heal?	GPT-5	Evaluator 2	62
Q7	How long will it take for my knee to heal?	GPT-5	Evaluator 3	48
Q7	How long will it take for my knee to heal?	GPT-5	Evaluator 4	34
Q7	How long will it take for my knee to heal?	Gemini 2.5 Pro	Evaluator 1	66
Q7	How long will it take for my knee to heal?	Gemini 2.5 Pro	Evaluator 2	60
Q7	How long will it take for my knee to heal?	Gemini 2.5 Pro	Evaluator 3	52
Q7	How long will it take for my knee to heal?	Gemini 2.5 Pro	Evaluator 4	43
Q7	How long will it take for my knee to heal?	Grok 4	Evaluator 1	62
Q7	How long will it take for my knee to heal?	Grok 4	Evaluator 2	56
Q7	How long will it take for my knee to heal?	Grok 4	Evaluator 3	54
Q7	How long will it take for my knee to heal?	Grok 4	Evaluator 4	45
Q7	How long will it take for my knee to heal?	DeepSeek-V3.2-Exp	Evaluator 1	57
Q7	How long will it take for my knee to heal?	DeepSeek-V3.2-Exp	Evaluator 2	62
Q7	How long will it take for my knee to heal?	DeepSeek-V3.2-Exp	Evaluator 3	51
Q7	How long will it take for my knee to heal?	DeepSeek-V3.2-Exp	Evaluator 4	36
Q8	Do I need surgery?	GPT-5	Evaluator 1	62
Q8	Do I need surgery?	GPT-5	Evaluator 2	67
Q8	Do I need surgery?	GPT-5	Evaluator 3	48

Question code	Question	Model	Evaluator	Total DISCERN score
Q8	Do I need surgery?	GPT-5	Evaluator 4	34
Q8	Do I need surgery?	Gemini 2.5 Pro	Evaluator 1	64
Q8	Do I need surgery?	Gemini 2.5 Pro	Evaluator 2	62
Q8	Do I need surgery?	Gemini 2.5 Pro	Evaluator 3	53
Q8	Do I need surgery?	Gemini 2.5 Pro	Evaluator 4	41
Q8	Do I need surgery?	Grok 4	Evaluator 1	62
Q8	Do I need surgery?	Grok 4	Evaluator 2	69
Q8	Do I need surgery?	Grok 4	Evaluator 3	54
Q8	Do I need surgery?	Grok 4	Evaluator 4	45
Q8	Do I need surgery?	DeepSeek-V3.2-Exp	Evaluator 1	64
Q8	Do I need surgery?	DeepSeek-V3.2-Exp	Evaluator 2	60
Q8	Do I need surgery?	DeepSeek-V3.2-Exp	Evaluator 3	51
Q8	Do I need surgery?	DeepSeek-V3.2-Exp	Evaluator 4	36
Q9	Do I need physical therapy?	GPT-5	Evaluator 1	64
Q9	Do I need physical therapy?	GPT-5	Evaluator 2	65
Q9	Do I need physical therapy?	GPT-5	Evaluator 3	56
Q9	Do I need physical therapy?	GPT-5	Evaluator 4	39
Q9	Do I need physical therapy?	Gemini 2.5 Pro	Evaluator 1	68
Q9	Do I need physical therapy?	Gemini 2.5 Pro	Evaluator 2	68
Q9	Do I need physical therapy?	Gemini 2.5 Pro	Evaluator 3	55
Q9	Do I need physical therapy?	Gemini 2.5 Pro	Evaluator 4	39
Q9	Do I need physical therapy?	Grok 4	Evaluator 1	54
Q9	Do I need physical therapy?	Grok 4	Evaluator 2	60
Q9	Do I need physical therapy?	Grok 4	Evaluator 3	62
Q9	Do I need physical therapy?	Grok 4	Evaluator 4	42
Q9	Do I need physical therapy?	DeepSeek-V3.2-Exp	Evaluator 1	72
Q9	Do I need physical therapy?	DeepSeek-V3.2-Exp	Evaluator 2	67
Q9	Do I need physical therapy?	DeepSeek-V3.2-Exp	Evaluator 3	53
Q9	Do I need physical therapy?	DeepSeek-V3.2-Exp	Evaluator 4	35
Q10	Are there any conservative options besides physical therapy?	GPT-5	Evaluator 1	64
Q10	Are there any conservative options besides physical therapy?	GPT-5	Evaluator 2	60
Q10	Are there any conservative options besides physical therapy?	GPT-5	Evaluator 3	51
Q10	Are there any conservative options besides physical therapy?	GPT-5	Evaluator 4	39
Q10	Are there any conservative options besides physical therapy?	Gemini 2.5 Pro	Evaluator 1	63
Q10	Are there any conservative options besides physical therapy?	Gemini 2.5 Pro	Evaluator 2	62
Q10	Are there any conservative options besides physical therapy?	Gemini 2.5 Pro	Evaluator 3	50

Question code	Question	Model	Evaluator	Total DISCERN score
Q10	Are there any conservative options besides physical therapy?	Gemini 2.5 Pro	Evaluator 4	39
Q10	Are there any conservative options besides physical therapy?	Grok 4	Evaluator 1	64
Q10	Are there any conservative options besides physical therapy?	Grok 4	Evaluator 2	65
Q10	Are there any conservative options besides physical therapy?	Grok 4	Evaluator 3	51
Q10	Are there any conservative options besides physical therapy?	Grok 4	Evaluator 4	49
Q10	Are there any conservative options besides physical therapy?	DeepSeek-V3.2-Exp	Evaluator 1	70
Q10	Are there any conservative options besides physical therapy?	DeepSeek-V3.2-Exp	Evaluator 2	70
Q10	Are there any conservative options besides physical therapy?	DeepSeek-V3.2-Exp	Evaluator 3	51
Q10	Are there any conservative options besides physical therapy?	DeepSeek-V3.2-Exp	Evaluator 4	40
Q11	Should I rest completely or keep exercising?	GPT-5	Evaluator 1	70
Q11	Should I rest completely or keep exercising?	GPT-5	Evaluator 2	60
Q11	Should I rest completely or keep exercising?	GPT-5	Evaluator 3	56
Q11	Should I rest completely or keep exercising?	GPT-5	Evaluator 4	39
Q11	Should I rest completely or keep exercising?	Gemini 2.5 Pro	Evaluator 1	66
Q11	Should I rest completely or keep exercising?	Gemini 2.5 Pro	Evaluator 2	64
Q11	Should I rest completely or keep exercising?	Gemini 2.5 Pro	Evaluator 3	60
Q11	Should I rest completely or keep exercising?	Gemini 2.5 Pro	Evaluator 4	43
Q11	Should I rest completely or keep exercising?	Grok 4	Evaluator 1	68
Q11	Should I rest completely or keep exercising?	Grok 4	Evaluator 2	64
Q11	Should I rest completely or keep exercising?	Grok 4	Evaluator 3	58
Q11	Should I rest completely or keep exercising?	Grok 4	Evaluator 4	44
Q11	Should I rest completely or keep exercising?	DeepSeek-V3.2-Exp	Evaluator 1	67
Q11	Should I rest completely or keep exercising?	DeepSeek-V3.2-Exp	Evaluator 2	64
Q11	Should I rest completely or keep exercising?	DeepSeek-V3.2-Exp	Evaluator 3	59
Q11	Should I rest completely or keep exercising?	DeepSeek-V3.2-Exp	Evaluator 4	36
Q12	What can I do for immediate pain relief?	GPT-5	Evaluator 1	66
Q12	What can I do for immediate pain relief?	GPT-5	Evaluator 2	64
Q12	What can I do for immediate pain relief?	GPT-5	Evaluator 3	56
Q12	What can I do for immediate pain relief?	GPT-5	Evaluator 4	39
Q12	What can I do for immediate pain relief?	Gemini 2.5 Pro	Evaluator 1	68
Q12	What can I do for immediate pain relief?	Gemini 2.5 Pro	Evaluator 2	64
Q12	What can I do for immediate pain relief?	Gemini 2.5 Pro	Evaluator 3	60
Q12	What can I do for immediate pain relief?	Gemini 2.5 Pro	Evaluator 4	37
Q12	What can I do for immediate pain relief?	Grok 4	Evaluator 1	62

Question code	Question	Model	Evaluator	Total DISCERN score
Q12	What can I do for immediate pain relief?	Grok 4	Evaluator 2	58
Q12	What can I do for immediate pain relief?	Grok 4	Evaluator 3	61
Q12	What can I do for immediate pain relief?	Grok 4	Evaluator 4	46
Q12	What can I do for immediate pain relief?	DeepSeek-V3.2-Exp	Evaluator 1	65
Q12	What can I do for immediate pain relief?	DeepSeek-V3.2-Exp	Evaluator 2	67
Q12	What can I do for immediate pain relief?	DeepSeek-V3.2-Exp	Evaluator 3	59
Q12	What can I do for immediate pain relief?	DeepSeek-V3.2-Exp	Evaluator 4	39
Q13	Will a knee brace or taping help?	GPT-5	Evaluator 1	66
Q13	Will a knee brace or taping help?	GPT-5	Evaluator 2	60
Q13	Will a knee brace or taping help?	GPT-5	Evaluator 3	56
Q13	Will a knee brace or taping help?	GPT-5	Evaluator 4	38
Q13	Will a knee brace or taping help?	Gemini 2.5 Pro	Evaluator 1	67
Q13	Will a knee brace or taping help?	Gemini 2.5 Pro	Evaluator 2	64
Q13	Will a knee brace or taping help?	Gemini 2.5 Pro	Evaluator 3	55
Q13	Will a knee brace or taping help?	Gemini 2.5 Pro	Evaluator 4	39
Q13	Will a knee brace or taping help?	Grok 4	Evaluator 1	62
Q13	Will a knee brace or taping help?	Grok 4	Evaluator 2	57
Q13	Will a knee brace or taping help?	Grok 4	Evaluator 3	57
Q13	Will a knee brace or taping help?	Grok 4	Evaluator 4	42
Q13	Will a knee brace or taping help?	DeepSeek-V3.2-Exp	Evaluator 1	72
Q13	Will a knee brace or taping help?	DeepSeek-V3.2-Exp	Evaluator 2	64
Q13	Will a knee brace or taping help?	DeepSeek-V3.2-Exp	Evaluator 3	59
Q13	Will a knee brace or taping help?	DeepSeek-V3.2-Exp	Evaluator 4	38
Q14	Why do I hear cracking or grinding (crepitus)?	GPT-5	Evaluator 1	55
Q14	Why do I hear cracking or grinding (crepitus)?	GPT-5	Evaluator 2	61
Q14	Why do I hear cracking or grinding (crepitus)?	GPT-5	Evaluator 3	56
Q14	Why do I hear cracking or grinding (crepitus)?	GPT-5	Evaluator 4	36
Q14	Why do I hear cracking or grinding (crepitus)?	Gemini 2.5 Pro	Evaluator 1	48
Q14	Why do I hear cracking or grinding (crepitus)?	Gemini 2.5 Pro	Evaluator 2	64
Q14	Why do I hear cracking or grinding (crepitus)?	Gemini 2.5 Pro	Evaluator 3	57
Q14	Why do I hear cracking or grinding (crepitus)?	Gemini 2.5 Pro	Evaluator 4	38
Q14	Why do I hear cracking or grinding (crepitus)?	Grok 4	Evaluator 1	52
Q14	Why do I hear cracking or grinding (crepitus)?	Grok 4	Evaluator 2	61
Q14	Why do I hear cracking or grinding (crepitus)?	Grok 4	Evaluator 3	56
Q14	Why do I hear cracking or grinding (crepitus)?	Grok 4	Evaluator 4	45
Q14	Why do I hear cracking or grinding (crepitus)?	DeepSeek-V3.2-Exp	Evaluator 1	55
Q14	Why do I hear cracking or grinding (crepitus)?	DeepSeek-V3.2-Exp	Evaluator 2	67
Q14	Why do I hear cracking or grinding (crepitus)?	DeepSeek-V3.2-Exp	Evaluator 3	55
Q14	Why do I hear cracking or grinding (crepitus)?	DeepSeek-V3.2-Exp	Evaluator 4	37
Q15	When can I return to my sport or running?	GPT-5	Evaluator 1	65

Question code	Question	Model	Evaluator	Total DISCERN score
Q15	When can I return to my sport or running?	GPT-5	Evaluator 2	60
Q15	When can I return to my sport or running?	GPT-5	Evaluator 3	56
Q15	When can I return to my sport or running?	GPT-5	Evaluator 4	37
Q15	When can I return to my sport or running?	Gemini 2.5 Pro	Evaluator 1	65
Q15	When can I return to my sport or running?	Gemini 2.5 Pro	Evaluator 2	67
Q15	When can I return to my sport or running?	Gemini 2.5 Pro	Evaluator 3	57
Q15	When can I return to my sport or running?	Gemini 2.5 Pro	Evaluator 4	41
Q15	When can I return to my sport or running?	Grok 4	Evaluator 1	59
Q15	When can I return to my sport or running?	Grok 4	Evaluator 2	61
Q15	When can I return to my sport or running?	Grok 4	Evaluator 3	56
Q15	When can I return to my sport or running?	Grok 4	Evaluator 4	42
Q15	When can I return to my sport or running?	DeepSeek-V3.2-Exp	Evaluator 1	65
Q15	When can I return to my sport or running?	DeepSeek-V3.2-Exp	Evaluator 2	72
Q15	When can I return to my sport or running?	DeepSeek-V3.2-Exp	Evaluator 3	55
Q15	When can I return to my sport or running?	DeepSeek-V3.2-Exp	Evaluator 4	38
Q16	What is the chance of this pain coming back?	GPT-5	Evaluator 1	63
Q16	What is the chance of this pain coming back?	GPT-5	Evaluator 2	67
Q16	What is the chance of this pain coming back?	GPT-5	Evaluator 3	56
Q16	What is the chance of this pain coming back?	GPT-5	Evaluator 4	38
Q16	What is the chance of this pain coming back?	Gemini 2.5 Pro	Evaluator 1	73
Q16	What is the chance of this pain coming back?	Gemini 2.5 Pro	Evaluator 2	64
Q16	What is the chance of this pain coming back?	Gemini 2.5 Pro	Evaluator 3	52
Q16	What is the chance of this pain coming back?	Gemini 2.5 Pro	Evaluator 4	42
Q16	What is the chance of this pain coming back?	Grok 4	Evaluator 1	60
Q16	What is the chance of this pain coming back?	Grok 4	Evaluator 2	64
Q16	What is the chance of this pain coming back?	Grok 4	Evaluator 3	53
Q16	What is the chance of this pain coming back?	Grok 4	Evaluator 4	43
Q16	What is the chance of this pain coming back?	DeepSeek-V3.2-Exp	Evaluator 1	62
Q16	What is the chance of this pain coming back?	DeepSeek-V3.2-Exp	Evaluator 2	72
Q16	What is the chance of this pain coming back?	DeepSeek-V3.2-Exp	Evaluator 3	57
Q16	What is the chance of this pain coming back?	DeepSeek-V3.2-Exp	Evaluator 4	38
Q17	How can I prevent PFPS from happening again?	GPT-5	Evaluator 1	67
Q17	How can I prevent PFPS from happening again?	GPT-5	Evaluator 2	67
Q17	How can I prevent PFPS from happening again?	GPT-5	Evaluator 3	51
Q17	How can I prevent PFPS from happening again?	GPT-5	Evaluator 4	36
Q17	How can I prevent PFPS from happening again?	Gemini 2.5 Pro	Evaluator 1	70
Q17	How can I prevent PFPS from happening again?	Gemini 2.5 Pro	Evaluator 2	62
Q17	How can I prevent PFPS from happening again?	Gemini 2.5 Pro	Evaluator 3	52
Q17	How can I prevent PFPS from happening again?	Gemini 2.5 Pro	Evaluator 4	42
Q17	How can I prevent PFPS from happening again?	Grok 4	Evaluator 1	59

Question code	Question	Model	Evaluator	Total DISCERN score
Q17	How can I prevent PFPS from happening again?	Grok 4	Evaluator 2	61
Q17	How can I prevent PFPS from happening again?	Grok 4	Evaluator 3	54
Q17	How can I prevent PFPS from happening again?	Grok 4	Evaluator 4	49
Q17	How can I prevent PFPS from happening again?	DeepSeek-V3.2-Exp	Evaluator 1	71
Q17	How can I prevent PFPS from happening again?	DeepSeek-V3.2-Exp	Evaluator 2	70
Q17	How can I prevent PFPS from happening again?	DeepSeek-V3.2-Exp	Evaluator 3	51
Q17	How can I prevent PFPS from happening again?	DeepSeek-V3.2-Exp	Evaluator 4	40

End of Supplementary File S2
